# Maternal Embryonic Leucine Zipper Kinase is Associated with Metastasis in Triple-negative Breast Cancer

**DOI:** 10.1158/2767-9764.CRC-22-0330

**Published:** 2023-06-20

**Authors:** Xuemei Xie, Gaurav B. Chauhan, Ramakrishna Edupuganti, Takahiro Kogawa, Jihyun Park, Moises Tacam, Alex W. Tan, Mohd Mughees, Fnu Vidhu, Diane D. Liu, Juliana M. Taliaferro, Mary Kathryn Pitner, Luke S. Browning, Ju-Hyeon Lee, François Bertucci, Yu Shen, Jian Wang, Naoto T. Ueno, Savitri Krishnamurthy, Gabriel N. Hortobagyi, Debu Tripathy, Steven J. Van Laere, Geoffrey Bartholomeusz, Kevin N. Dalby, Chandra Bartholomeusz

**Affiliations:** 1 Section of Translational Breast Cancer Research, Houston, Texas.; 2 Department of Breast Medical Oncology, The University of Texas MD Anderson Cancer Center, Houston, Texas.; 3 Division of Chemical Biology & Medicinal Chemistry, College of Pharmacy, The University of Texas at Austin, Austin, Texas.; 4 Department of Biostatistics, The University of Texas MD Anderson Cancer Center, Houston, Texas.; 5 Predictive Oncology Laboratory, Marseille Research Cancer Center, INSERM U1068, CNRS U7258, Institut Paoli-Calmettes, Aix Marseille University, 13009 Marseille, France.; 6 Current Institution: Cancer Biology Program, University of Hawai'i Cancer Center, Honolulu, Hawaii, USA.; 7 Department of Pathology, University of Texas MD Anderson Cancer Center, Houston, Texas.; 8 Division of Pathology and Laboratory Medicine, The University of Texas MD Anderson Cancer Center, Houston, Texas.; 9 Center for Oncological Research, Integrated Personalized and Precision Oncology Network, University of Antwerp, Antwerp, Wilrijk.; 10 Department Oncology, KU Leuven, Leuven, Belgium.; 11 Experimental Therapeutics, The University of Texas MD Anderson Cancer Center, Houston, Texas.

## Abstract

Triple-negative breast cancer (TNBC) has high relapse and metastasis rates and a high proportion of cancer stem-like cells (CSC), which possess self-renewal and tumor initiation capacity. MELK (maternal embryonic leucine zipper kinase), a protein kinase of the Snf1/AMPK kinase family, is known to promote CSC maintenance and malignant transformation. However, the role of MELK in TNBC metastasis is unknown; we sought to address this in the current study. We found that *MELK* mRNA levels were higher in TNBC tumors [8.11 (3.79–10.95)] than in HR^+^HER2^−^ tumors [6.54 (2.90–9.26)]; *P <* 0.001]. In univariate analysis, patients with breast cancer with high-*MELK*–expressing tumors had worse overall survival (*P <* 0.001) and distant metastasis-free survival (*P <* 0.01) than patients with low-*MELK*–expressing tumors. In a multicovariate Cox regression model, high MELK expression was associated with shorter overall survival after adjusting for other baseline risk factors. MELK knockdown using siRNA or MELK inhibition using the MELK inhibitor MELK-In-17 significantly reduced invasiveness, reversed epithelial-to-mesenchymal transition, and reduced CSC self-renewal and maintenance in TNBC cells. Nude mice injected with CRISPR MELK-knockout MDA-MB-231 cells exhibited suppression of lung metastasis and improved overall survival compared with mice injected with control cells (*P* < 0.05). Furthermore, MELK-In-17 suppressed 4T1 tumor growth in syngeneic BALB/c mice (*P* < 0.001). Our findings indicate that MELK supports metastasis by promoting epithelial-to-mesenchymal transition and the CSC phenotype in TNBC.

**Significance:**

These findings indicate that MELK is a driver of aggressiveness and metastasis in TNBC.

## Introduction

Breast cancer is the most common cancer in women and is the second leading cause of cancer deaths in the United States. Triple-negative breast cancer (TNBC), which lacks estrogen receptor (ER), progesterone receptor (PR), and HER2, accounts for 15%–20% of all breast cancer cases (1). TNBC is highly invasive, and approximately 46% of patients with TNBC will have distant metastasis (2). Among breast cancer subtypes defined by ER, PR, and HER2 status, TNBC has the worst survival outcomes (3), which is mainly due to its high recurrence after surgery (30%–40% of patients) and limited targeted therapies (4, 5).

TNBC is characterized by epithelial-to-mesenchymal transition (EMT), which leads to the generation of breast cancer cells with stem cell–like characteristics (6). The induction of EMT in immortalized, nontumorigenic human mammary epithelial cells resulted in acquisition of the CD44^+^/CD24^–^ phenotype, a characteristic of breast cancer stem-like cells (CSC). Microarray analysis of breast cancer tumors showed that the gene expression profile of the CD44^+^/CD24^–^ fraction resembled that of stem cell–like cells, and this subpopulation was able to form mammospheres *in vitro* (6). Breast cancer cells with the CD44^+^/CD24^−^ phenotype were enriched for tumor-initiating CSCs and were highly invasive (6–8), and the prevalence of CD44^+^/CD24^−^ tumor cells in breast cancer may favor distant metastasis (9). Compared with other breast cancer subtypes, TNBC has a higher proportion of CSCs and is linked to EMT, two critical features associated with breast cancer progression, metastasis, and recurrence (10). Patients with TNBC have a very poor prognosis owing to the tendency of TNBC to relapse and metastasize, partially attributed to CSCs (11–13). Given the importance of CSCs in tumor initiation, metastasis, and therapy resistance, many efforts are underway to identify CSC-targeting compounds (14).

MELK (maternal embryonic leucine zipper kinase), a member of the Snf1/AMPK family of kinases, is overexpressed in various cancers, including breast cancer (15), and has been shown to be involved in CSC maintenance, malignant transformation, and cancer cell proliferation (16). Unchecked MELK activity has been implicated in the onset of various cancers (17–20). Recently, an analysis of The Cancer Genome Atlas data showed that MELK is in the top 1% of overexpressed genes in breast cancer, and its expression is eight times as high in breast tumors as in normal breast tissues (17). MELK is frequently upregulated in basal-like breast cancer compared with the other subtypes of breast cancer (17), and elevated MELK expression in breast cancer tissue is associated with shorter patient survival (19). In patients with early-stage node-negative disease, elevated MELK expression levels were associated with increased local recurrence rates after radiotherapy (21). These findings suggest that deeper exploration into the relationship between MELK and breast cancer formation and progression is warranted.

EMT is an essential developmental process by which cells of epithelial origin lose epithelial characteristics and polarity and acquire a mesenchymal phenotype with fibroblast-like morphology. In breast cancer, this process is associated with increased invasiveness, metastatic capability, and drug resistance (22–24). It has been proposed that EMT-like processes allow tumor cells to disassemble and migrate to distant tissue or organ sites (22, 23). EMT has been implicated in the aggressiveness of basal-like breast cancer (25). A tissue microarray analysis of 479 breast carcinomas showed an upregulation of mesenchymal markers and reduction of epithelial markers in basal-like breast tumors (25). However, how the EMT process relates to MELK function and whether this relationship impacts TNBC metastasis remains unknown.

In the current study, we sought to determine the role of MELK in TNBC metastasis. We found that high MELK expression was associated with worse outcomes in patients with breast cancer. In addition, overexpression of MELK in TNBC cells resulted in an increase in mammosphere formation, CD44^+^/CD24^−^ and ALDH1^+^ subpopulations, cell growth, invasion, and migration, whereas knockdown of MELK by siRNA or inhibition of MELK activity using a novel small-molecule MELK inhibitor, MELK-In-17 (also termed compound 17; ref. 26), suppressed these activities and reduced expression of mesenchymal markers. Furthermore, MELK CRISPR knockout (KO) suppressed lung metastasis, and treatment with MELK-In-17 suppressed tumor growth in mouse models. Our findings indicate that MELK supports a CSC phenotype, EMT, and metastasis in TNBC.

## Materials and Methods

### Patient Data

For a retrospective patient analysis, we used the World IBC Consortium dataset, which contains data from 389 patients with inflammatory breast cancer (IBC) and non-IBC at three institutions: The University of Texas MD Anderson Cancer Center, Houston, TX (83 patients; years 2000 to 2005); General Hospital Sint-Augustinus, Antwerp, Belgium (96 patients; years 1996 to 2009); and Institut Paoli-Calmettes, Marseille, France (210 patients; years 1998 to 2008). The dataset includes clinical and tumor characteristics and gene expression profiles for each patient. All patients gave written informed consent for voluntary participation. The Institutional Review Boards of all three participating centers approved our use of the data in the current study. Our analysis included data from 314 patients with stage I, II, or III primary breast cancer, after excluding 50 patients with metastatic breast disease and 25 with unknown metastatic status. All patients received multidisciplinary treatment according to the guidelines of each institution. Some of the patients (*n* = 87) received neoadjuvant systemic treatment, with the addition of hormone treatment for patients whose tumors were ER-positive or trastuzumab for patients with HER2 overexpression and/or amplification, if clinically available. Chemotherapy regimens containing taxane and anthracycline were administered at physician discretion.

### Gene Expression Profiling

Using the World IBC Consortium dataset, we assessed the association between MELK gene expression and molecular subtype of breast cancer or patient survival outcomes. We determined hormone receptor (HR) and HER2 positivity status using gene expression profiling (HR-positive was defined as ER- and/or PR-positive). Initially, RNA extraction and hybridization onto Affymetrix GeneChip (HGU133-series) were conducted as described previously (27). Gene expression data were normalized with the MAS 5.0 algorithm, mean-centered to 600, and log_2_-transformed. Immunohistochemistry (IHC) staining results for ER/PR/HER2 expression or HER2 FISH ratios were not available for 11 patients in the dataset; therefore, we used mRNA gene expression data for *ESR1* (probe set id; 205225_at) to determine ER receptor status, *PgR* (208305_at) for PR status, and *ERBB2* (216836_s_at) for HER2 status. We constructed ROC curves to measure the predictive accuracy of the logistic regression models, including *ESR1*, *PgR*, and *ERBB2* mRNA expression levels. Normalized mRNA expression levels of *ESR1* >10.18, *PgR* >2.91, and *ERBB2* >12.54 were considered amplified (28). Tumor grade was determined using the genomic grade index. We identified 173 patients with HR^+^HER2^−^, 16 with HR^+^HER2^+^, 41 with HR^−^HER2^+^, and 84 with TNBC. Statistical analysis was performed using BRB-Array Tools version 3.9.0α software and R software version 2.7.2. For consistent quantification of the World IBC Consortium array, we used the fRMA algorithm, analyzing the array individually or in small batches; hence we combined the data to normalize and quantify the whole dataset. Clinicopathologic characteristics of the patients in our analysis according to breast cancer subtype are presented in Table 1.

**TABLE 1:** Clinicopathologic characteristics of patients in the World IBC Consortium dataset whose data were used for our analysis (*n* = 314)

	No. (%)					
Covariate	All *n* = 314	TNBC *n* = 84	HR^+^HER2^−^ *n* = 173	HR^−^HER2^+^ *n* = 41	HR^+^HER2^+^ *n* = 16	*P*
Tumor						<0.0001
IBC	86 (27.4)	35 (40.7)	26(30.2)	20 (23.3)	5 (5.8)	
Non-IBC	228 (72.6)	49 (21.5)	147(64.5)	21 (9.2)	11 (4.8)	
Histologic characteristics						0.1139
Ductal	259 (82.5)	76 (29.3)	133 (51.4)	37 (14.3)	13 (5.0)	
Lobular	26 (8.3)	4 (15.4)	20 (76.9)	1 (3.8)	1 (3.8)	
Others	29 (9.2)	4 (13.8)	20 (69.0)	3 (10.3)	2 (6.9)	
Clinical stage						0.0002
I	65 (20.7)	11 (16.9)	48 (73.8)	4 (6.2)	2 (3.1)	
II	97 (30.9)	20 (20.6)	63 (64.9)	11 (11.3)	3 (3.1)	
III	152 (48.4)	53 (34.9)	62 (40.8)	26 (17.1)	11 (7.2)	
Lymphatic invasion						0.1373
Unknown	125					
0	108 (57.1)	32 (29.6)	58 (53.7)	13 (12.0)	5 (4.6)	
1	81 (42.9)	22 (27.2)	34 (42.0)	17 (21.0)	8 (9.9)	
Genomic grade index						<0.0001
GR1	115 (36.6)	15 (13.0)	90 (78.3)	6 (5.2)	4 (3.5)	
GR3	199 (63.4)	69 (34.7)	83 (41.7)	35 (17.6)	12 (6.0)	
Nuclear grade						<0.0001
Unknown	4					
1	50 (16.1)	4 (8.0)	46 (92.0)	0 (0.0)	0 (0.0)	
2	106 (34.2)	20 (18.9)	74 (69.8)	6 (5.7)	6 (5.7)	
3	154 (49.7)	58 (37.7)	51 (33.1)	35 (22.7)	10 (6.5)	
Neoadjuvant chemotherapy						0.0001
Unknown	11					
No	96 (31.7)	24 (25.0)	66 (68.8)	2 (2.1)	4 (4.2)	
Yes	207 (68.3)	59 (28.5)	100 (48.3)	37 (17.9)	11 (5.3)	
Hormone treatment						<0.0001
Unknown	78					
No	111 (47.0)	45 (40.5)	44 (39.6)	20 (18.0)	2 (1.8)	
Yes	125 (53.0)	14 (11.2)	97 (77.6)	6 (4.8)	8 (6.4)	

NOTE: *P* values reflect comparisons of covariate levels among the subgroups. Abbreviations: HER2, human growth factor receptor 2; IBC, inflammatory breast cancer; HR, hormone receptor (estrogen receptor or progesterone receptor); TNBC, triple-negative breast cancer.

### Reagents, Cell Lines, and Culture Conditions

MELK-In-17 (*K*_i_ = 0.39 nmol/L) was prepared as described previously (26). MDA-MB-231, HCC70, BT-549, and SUM159 human TNBC cells and 4T1 murine TNBC cells were purchased from ATCC. SUM149 TNBC cells were purchased from Asterand. All the human cell lines were originally obtained from female patients, and 4T1 is a mammary carcinoma originally derived from a spontaneously arising mammary tumor in BALB/cfC3H mice. MDA-MB-231 cells were maintained in DMEM/F12 medium (Life Technologies Inc) supplemented with FBS (10%) and antibiotic/antimycotic (1%). HCC70, BT-549, and 4T1 cells were maintained in RPMI1640 medium (Life Technologies Inc) supplemented with FBS (10%) and antibiotic/antimycotic (1%). SUM149 and SUM159 cells were maintained in Ham F-12 medium (Life Technologies Inc) supplemented with FBS (5%), antibiotic/antimycotic (1%), insulin (5 μg/mL), and hydrocortisone (1 μg/mL). All cell lines used in the current study were authenticated (BT-549 by September 30, 2022; HCC70 by October 16, 2017; MDA-MB-231 by August 27, 2018; SUM149 by August 27, 2018; and SUM159 by May 24, 2017) by the Characterized Cell Line Core Facility at The University of Texas MD Anderson Cancer Center (Houston, TX) using a short tandem repeat method based on primer extension to detect single base deviations. 4T1 murine cells were not authenticated. These cell lines were tested for *Mycoplasma* using the MycoAlert *Mycoplasma* Detection kit (catalog no.: LT07-218; Lonza Bioscience), and the last tested was done in November 2022. All cell lines were confirmed to be free of *Mycoplasma* and were used for less than 30 passages after being thawed from the frozen stocks.

### CRISPR KO of MELK in MDA-MB-231 Cells

A Cas9-expressing stable cell line was generated by transfecting MDA-MB-231 cells with a Cas9 LentiCRISPR construct containing the blasticidin selection marker (Life Technologies Inc). Following transfection with the Cas9 LentiCRISPR construct, single Cas9-expressing stable clones were selected using blasticidin and confirmed by Western blot analysis. Then, three customer-designed *MELK* sequence–specific guide RNAs (IVTgRNA; Life Technologies Inc) were transfected into Cas9-expressing MDA-MB-231 stable cells. For transfection, 90 μL of Cas9-expressing MDA-MB-231 stable clones were seeded into a 96-well plate containing 10 μL of DharmaFect 4 (Dharmacon Lafayette) and IVTgMELK complex. Three separated transfections were performed using each of the three IVTMLKgRNA complexes, resulting in 21 ng gRNA and 10,000 cells per well. At 48 hours after transfection, cells were expanded into a 24-well plate and cultured until reaching confluency of 80% to 90%. Single clones were obtained by seeding 100 μL of a 10-mL cell suspension containing 100 cells and then expanded and used for Western blot screening for MELK KO and generation of a cell bank.

### siRNA Transfection

Cells (1 × 10^6^) were transfected with scrambled siRNA (SCR) control, siMELK#1, or siMELK#2 (Sigma-Aldrich) at a final siRNA concentration of 4 μmol/L using the Neon Transfection System (Life Technologies Inc).

### Plasmid Transfection

Cells (1 × 10^6^) were transfected with 8 μg of the HA-DDK-pcDNA3 empty vector (OriGene) or the HA-DDK-pcDNA3 vector carrying wild-type (WT) *MELK* (OriGene) or kinase-dead (D150A; KD) *MELK* using the Neon Transfection System.

### Quantitative Reverse Transcriptase PCR

Total RNA was extracted from the cells using the miRNeasy mini kit (Sigma-Aldrich) according to the manufacturer's instructions. One-step quantitative PCR reactions were performed using the SYBR green quantitative PCR kit (Bio-Rad Laboratories) with a pair of *MELK* primers [5′-GAAGGCTCGGGGAAAACCAG-3′ (forward) and 5′-TGTCTGTGAATGGGGTAGCA-3′ (reverse)] and a pair of *GAPDH* primers [5′-CCATGAGAAGTATGACAACAGCC-3′ (forward) and 5′-CCTTCCACGATACCAAAGTTG-3′ (reverse)]. mRNA of the human housekeeping gene *GAPDH* was used as a normalization control. mRNA levels of *MELK* were normalized to the mRNA levels of *GAPDH*, and the fold induction of *MELK* mRNA was calculated on the basis of the *MELK* mRNA level in SCR-treated control cells. Quantification of murine EMT marker mRNA levels is provided in Supplementary Materials and Methods.

### Western Blot Analysis

Western blot analysis was performed as described previously (29). Proteins of interest were probed using the following primary antibodies (1:1,000 dilution) purchased from Cell Signaling Technology or other suppliers as indicated: MELK (AF4820; R&D Systems), fibronectin (610078, 1:500 dilution; BD Transduction Laboratories), vimentin (5741s), E-cadherin (610181; BD Transduction Laboratories), N-cadherin (4061s), β-catenin (9581s), snail (sc-10433; Santa Cruz Biotechnology Inc), α-tubulin (T9026; Sigma-Aldrich), and β-actin (A5316; Sigma-Aldrich). Secondary antibodies were horseradish peroxidase–conjugated IgG (1:10,000 dilution; Life Technologies Inc) for chemiluminescent signal detection and the corresponding Alexa Fluor-conjugated IgG (1:5,000 dilution; Life Technologies Inc) for fluorescence signal detection.

### Cell Proliferation Assay

The effect of MELK knockdown on cell proliferation was determined using the trypan blue exclusion assay (30). The antiproliferation efficacy of MELK-In-17 was determined using the CellTiter-Blue Viability Assay (31). Additional details are provided in Supplementary Materials and Methods.

### Clonogenic Assay

The effects of WT or KD MELK overexpression on TNBC cell growth were determined using the clonogenic assay (32). Additional details are provided in Supplementary Materials and Methods.

### Anchorage-independent Growth Evaluation

The effects of MELK KO or MELK-In-17 treatment on TNBC cell growth were determined using the anchorage-independent growth assay (33). Additional details are provided in Supplementary Materials and Methods.

### Cell Migration and Invasion Assays

The effects of MELK knockdown, WT or KD MELK overexpression, or MELK-In-17 treatment on migration and invasion of TNBC cells were determined using migration and invasion assays (34). Additional details are provided in Supplementary Materials and Methods.

### Mammosphere Assay

The effects of MELK knockdown or KO, WT or KD MELK overexpression, or MELK-In-17 treatment on CSC self-renewal were determined using the mammosphere formation assay (34). Additional details are provided in Supplementary Materials and Methods.

### CSC Subpopulation Analysis

The effects of MELK knockdown or MELK-In-17 treatment on CD44^+^/CD24^−^ subpopulations and aldehyde dehydrogenase (ALDH) activity were determined by flow cytometry (34). Additional details are provided in Supplementary Materials and Methods.

### *In Vivo* Experimental Lung Metastasis

Cas9-p15 control or two MELK KO MDA-MB-231-Luc-GFP stable cell lines (1 × 10^6^ in 0.1 mL PBS) were injected into the tail vein of 6 to 8 weeks old female athymic nude mice. Metastatic tumors were measured weekly for 7 weeks using an IVIS 100 Imaging System (Xenogen Corporation). Before imaging, mice were injected intraperitoneally with d-Luciferin (150 mg/kg body weight, Caliper Life Sciences). Five minutes later, images were taken with the mice under anesthesia with isoflurane. Images and amounts of bioluminescent signals were analyzed using Living Image Software (Xenogen). At week 7, after the last IVIS imagining, mice were euthanized, and lung tissues were removed from each mouse for IHC analyses. Animal studies were approved by the institutional animal care and use committee of MD Anderson Cancer Center (00001235-RN01). Animal care and use were per Institutional and National Institutes of Health guidelines.

### Treatment with MELK-in-17 in a TNBC Xenograft Model

Murine 4T1 TNBC cells in log-phase growth were harvested and resuspended in PBS. Cells (1 × 10^4^) in 0.2 mL of PBS were injected under aseptic conditions into the mammary fat pads of BALB/c mice (Harlan Laboratories Inc). When tumor size reached 75–150 mm^3^, mice were randomly divided into three groups (10 mice/group) and treated with vehicle or MELK-In-17 at 5 or 10 mg/kg via intraperitoneal injections daily for 25 days. Tumor size was measured twice per week. Tumor volume (mm^3^) was calculated, and changes in tumor volumes were tested for statistical significance using the Mann–Whitney test. Animal care and use were per Institutional and NIH guidelines. Animal studies were approved by the institutional animal care and use committee of MD Anderson Cancer Center (00001235-RN01). Animal care and use were per Institutional and National Institutes of Health guidelines.

### Microarray Analysis

To identify the downstream targets of MELK, we performed Affymetrix Genechip microarray using parental, Cas9-p15 control, and two MELK KO (C3 and C28) MDA-MB-231 cells. In brief, the cells (1 × 10^6^ cells) were cultured in 10-cm plates for 48 hours, followed by RNA extraction using the Invitrogen PureLink RNA Mini Kit (Thermo Fisher Scientific). RNA expression was measured using Human Transcriptome 2.0 Array (Affymetrix Inc) at the Sequencing and Microarray Facility at MD Anderson according to standard Affymetrix protocols. Differential gene expression profile analysis was performed (see Supplementary Methods and Materials for details).

### Statistical Analysis

Clinical endpoints included overall survival (OS), progression-free survival (PFS), and distant metastasis-free survival (DMFS) from the date of diagnosis. Data were summarized using standard descriptive statistics and frequency tabulation. Comparisons of continuous variables among HR/HER2 subgroups were performed using the Kruskal–Wallis test. Associations between categorical variables were assessed via cross-tabulation and the *χ*^2^ test or Fisher exact test. Time-to-event endpoints were estimated using the Kaplan–Meier method, and comparisons between or among patient characteristic groups were assessed using the log-rank test. Multicovariate Cox proportional hazards models were applied to assess the effect of covariates of interest on time-to-event endpoints, adjusting for other covariates. To assess survival outcomes by *MELK* mRNA levels, we divided the patient cohort into two groups based on the median *MELK* mRNA expression level (i.e., *MELK* < 7 and *MELK* ≥ 7). All computations were carried out using SAS 9.3 (SAS Institute Inc) and Splus 8.2 (TIBCO Software Inc). Statistical significance of the *in vitro* and *in vivo* results was assessed using a two-tailed Student *t* test. *P* < 0.05 was considered statistically significant. Power analysis for the *in vivo* studies was conducted on the basis of comparison of the primary endpoint (tumor size) between treatment and vehicle groups. Given 10 mice/group, we had at least 80% power to detect an effect size of 1.33 in mean difference of tumor size between two groups, using a two-sample *t* test at a significance level of 0.05.

### Data Availability

The microarray data generated in this study are publicly available in Gene Expression Omnibus at GSE227774.

## Results

### High MELK Expression Levels Correlate with Worse Outcomes in Breast Cancer

**FIGURE 1:**
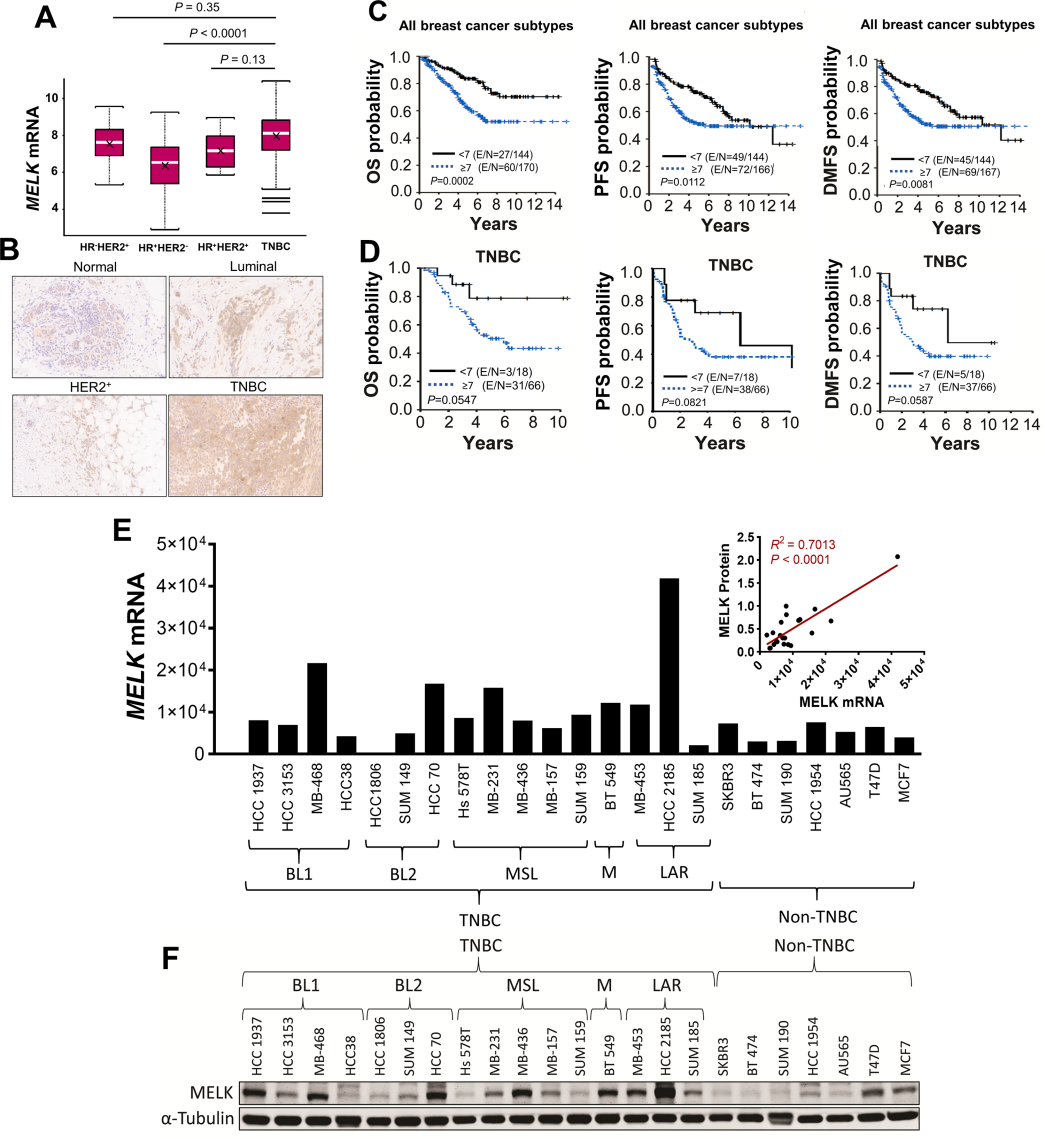
Clinical relevance of MELK in breast cancer and expression of MELK in breast cancer cell lines. **A,***MELK* mRNA levels in HR^−^HER2^+^, HR^+^HER2^−^, HR^+^HER2^+^, and TNBC tumors in the World Consortium IBC dataset, analyzed by multiple comparison using a general linear model. Data are presented as mean ± SD. – represents median, X represents mean. **B,** IHC images (200× magnification) showing MELK protein expression in normal epithelial tissue and luminal, HER2^+^, and TNBC breast tumors. Correlation between *MELK* mRNA levels (<7 or ≥7) and OS, PFS, and DMFS in patients in the overall cohort (**C**) and among those with TNBC (**D**), determined by the Kaplan–Meier method. *MELK* mRNA levels (**E**) and protein levels (**F**) in TNBC and non-TNBC cells. Inset, correlation between *MELK* mRNA and protein levels in TNBC cell lines. In F, α-tubulin was used as a loading control. *MELK* mRNA levels were determined using quantitative reverse transcriptase PCR.

We examined the clinical relevance of MELK using samples from 314 patients with IBC and non-IBC breast cancer with a median follow-up time of 5.2 years. Patient characteristics are summarized in Table 1. The median patient age was 54 years (range, 24–89 years). Of the patients with IBC, 40.7% had TNBC, whereas among the patients with non-IBC, 21.5% had TNBC. Among all patients in the cohort, 48.4% had stage III disease, 30.9% had stage II disease, and 20.7% had stage I disease; 82.5% of tumors had ductal histologic characteristics, and 49.7% had nuclear grade III. *MELK* mRNA expression levels were significantly higher in TNBC tumors [8.11 (3.79–10.95)] than in HR^+^HER2^−^ tumors [6.54 (2.90–9.26); *P <* 0.0001; Fig. 1A], and no difference in *MELK* mRNA levels was found between TNBC tumors and HR^+^HER2^+^ [7.17 (5.85–8.97)] or HR^−^HER2^+^ [7.62 (5.32–9.57)] tumors (Fig. 1A). Furthermore, MELK protein expression levels were high in TNBC tumors compared with normal epithelial tissue or luminal and HER2^+^ breast tumors (Fig. 1B). In univariate analysis, patients with breast cancer with tumors expressing high mRNA levels of *MELK* had significantly shorter 5-year OS (62.7% vs. 84.3%; *P* = 0.0002), PFS (51.8% vs. 73.3%; *P* = 0.0112), and DMFS (53.5% vs. 75.3%; *P* = 0.0081) than patients with tumors expressing low mRNA levels of *MELK* (Fig. 1C; Table 2). In a multicovariate Cox regression model, high *MELK* mRNA expression did not have independent prognostic value for PFS (*P* = 0.3721) or DMFS (*P* = 0.2853); however, compared with low *MELK* mRNA expression (<7), high *MELK* mRNA expression (≥7) was associated with shorter OS (HR = 1.791; 95% CI = 1.109–2.894; *P* = 0.0172), adjusted for tumor stage, IBC status, and TNBC status and stratified by the study centers (Table 3). In patients with TNBC (*n* = 84), high *MELK* mRNA expression showed marginally significant trend for poor OS (*P* = 0.0547), PFS (*P* = 0.0821), or DMFS (*P* = 0.0587; Fig. 1D). This nonsignificant finding may be due to the limited number of patients in this subgroup. Given that high levels of *MELK* mRNA expression were observed in TNBC patient tumors and TNBC cell lines and that TNBC has characteristics of CSC and EMT, we investigated the role of MELK in regulating TNBC metastasis in preclinical models.

**TABLE 2:** Univariate Cox regression model for OS, PFS, and DMFS among patients with breast cancer (World IBC Consortium dataset; *n* = 314)

Endpoint	Time	Event-free survival rate	95% CI	*P*
PFS				0.0112
*MELK* <7	2 years	83.6	76.3–88.8	
	5 years	73.3	64.7–80.1	
*MELK* ≥7	2 years	72.5	64.9–78.8	
	5 years	51.8	43.2–59.8	
DMFS				0.0081
*MELK* <7	2 years	85.0	77.9–89.9	
	5 years	75.3	66.8–82.0	
*MELK* ≥7	2 years	74.5	67.0–80.6	
	5 years	53.5	44.8–61.5	
OS				0.0002
*MELK* <7	2 years	94.2	88.7–97.1	
	5 years	84.3	76.3–89.7	
*MELK* ≥7	2 years	87.6	81.4–91.8	
	5 years	62.7	53.9–70.4	

NOTE: *P* values indicate differences between *MELK* <7 and *MELK* ≥7 groups for 2-year and 5-year outcomes. Abbreviations: *MELK*, maternal embryonic leucine zipper kinase mRNA level.

**TABLE 3:** Multicovariate Cox regression model for OS, PFS, and DMFS among patients with breast cancer (World IBC Consortium dataset; *n* = 314)

	OS			PFS			DMFS		
Parameter	HR	95% CI	*P*	HR	95% CI	*P*	HR	95% CI	*P*
Tumor type									
Non-IBC stage I vs. IBC stage III	0.255	0.112–0.581	0.0012	0.251	0.123–0.510	0.0001	0.260	0.123–0.549	0.0004
Non-IBC stage II vs. IBC stage III	0.720	0.412–1.256	0.2471	0.628	0.380–1.036	0.0688	0.693	0.411–1.168	0.1690
Non-IBC stage III vs. IBC stage III	0.630	0.315–1.258	0.1901	0.854	0.503–1.449	0.5579	0.896	0.520–1.544	0.6926
TNBC vs. non-TNBC	1.687	1.061–2.683	0.0271	1.630	1.087–2.445	0.0181	1.630	1.074–2.472	0.0216
*MELK* ≥7 vs. <7	1.791	1.109–2.894	0.0172	1.193	0.810–1.756	0.3721	1.243	0.834–1.854	0.2853

Abbreviations: IBC, inflammatory breast cancer; *MELK*, maternal embryonic leucine zipper kinase mRNA level; TNBC, triple-negative breast cancer.

### MELK is Highly Expressed in TNBC Cells and Promotes TNBC Cell Growth, Migration, and Invasion

Because high MELK expression was associated with a high risk of death and short OS in patients with breast cancer, we hypothesized that MELK promotes the growth of human breast cancer cells. To test this hypothesis, we first assessed MELK expression in 23 human breast cancer cell lines, of which 16 were TNBC and seven were non-TNBC. In agreement with our findings from the breast cancer patient dataset (Fig. 1A), we found that 10 of 16 TNBC cell lines (62%), compared with two of eight non-TNBC cell lines (25%), expressed high mRNA levels of *MELK* (Fig. 1E), which positively correlated with MELK protein levels (Fig. 1F).

**FIGURE 2:**
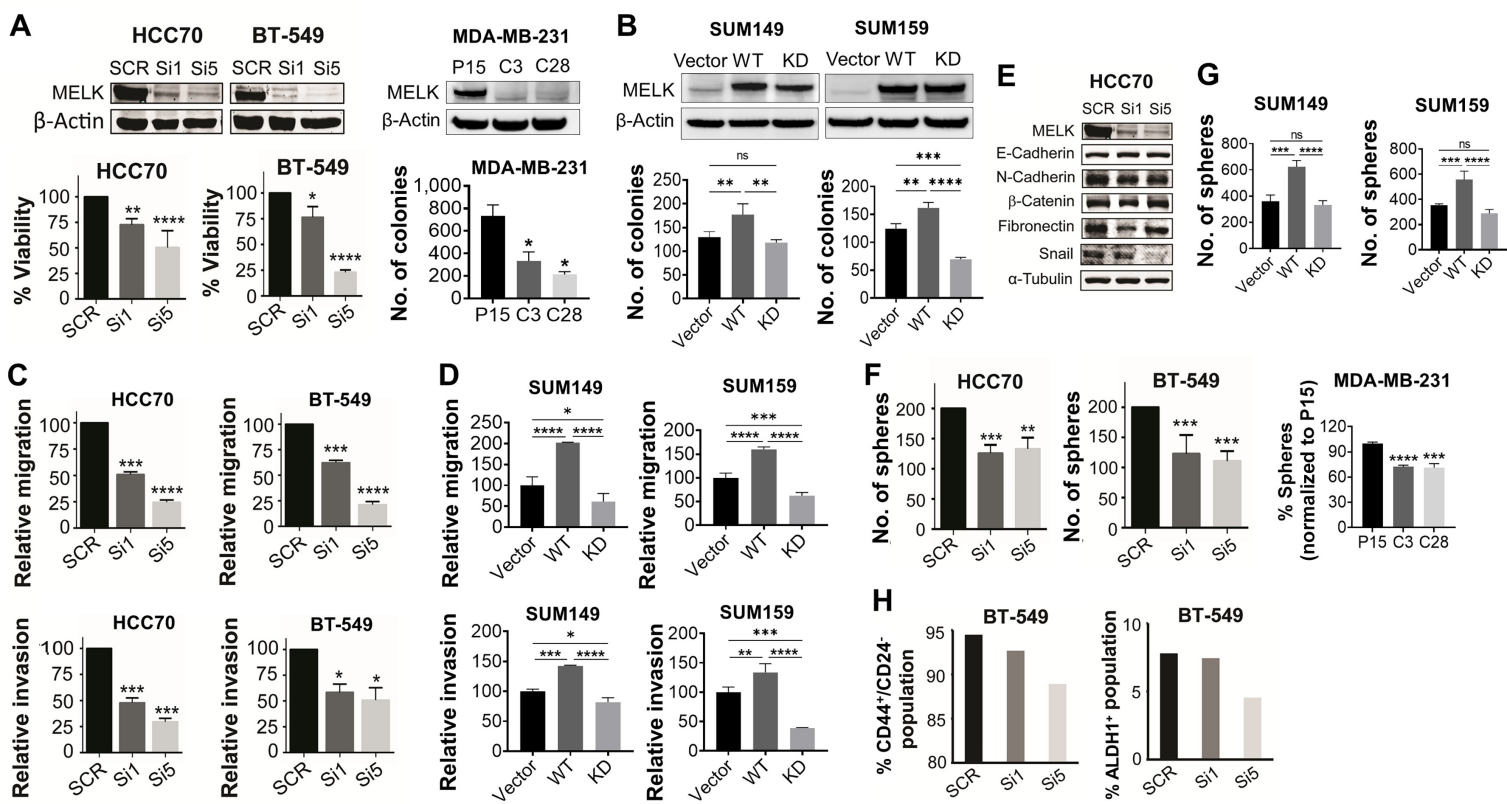
Effect of MELK knockdown or overexpression on proliferation, motility, EMT, and CSC properties in TNBC cells. Effect of MELK knockdown or KO (**A**) or overexpression (**B**) on proliferation and anchorage-independent growth of TNBC cells. HCC70 and BT-549 cells (A, bottom left) were treated with two different siMELK sequences for 48 hours. MELK expression was then determined using Western blot analysis (top), and cell viability was determined using trypan blue exclusion assay (bottom left). In MELK KO MDA-MB-231 cells (A, bottom right), anchorage-independent growth was determined using soft-agar assay at week 3 following incubation. β-Actin was used as a loading control for Western blot analysis. SUM149 and SUM159 cells (B) were transfected with plasmids encoding WT *MELK* gene or KD *MELK* gene and cultured for 48 hours. MELK expression was then determined using Western blot analysis (top), and cell growth was determined using anchorage-independent growth assay (bottom). β-Actin was used as a loading control for Western blot analysis. Effect of MELK knockdown (**C**) or overexpression (**D**) on migration and invasion of TNBC cells. Cells were transfected with two different siMELK sequences for 72 hours or plasmids encoding WT *MELK* gene or KD *MELK* gene for 48 hours and then assayed for migration and invasion. **E,** Effect of MELK knockdown on expression of EMT epithelial and mesenchymal markers in TNBC cells. Cells were treated with two different siMELK sequences for 48 hours, and then MELK expression was determined using Western blot analysis. α-Tubulin was used as a loading control. Effect of knockdown or KO (**F**) or overexpression (**G**) of MELK on mammosphere formation in TNBC cells. Cells were transfected with two different siMELK sequences or plasmids encoding WT *MELK* gene or KD *MELK* gene and 48 hours later were seeded for mammosphere formation. MELK KO clones were cultured for 7 days and then analyzed for mammosphere formation. **H,** Effect of MELK knockdown on CD44^+^/CD24^−^ and ALDH1^+^ subpopulations in TNBC cells. Cells were treated with two different siMELK sequences and 48 hours later subjected to flow cytometry analysis. In A and F, P15 indicates Cas9-p15 control cells; C3, MELK KO C3 clones; and C28, MELK KO C28 clones. In A–D, G, and F, data are presented as mean ± standard deviation. *, *P* < 0.05; **, *P* < 0.01; ***, *P* < 0.001; ****, *P* < 0.0001.

To investigate the biological role of MELK in TNBC, we used cells representing the heterogeneity of TNBC: HCC70 (basal-like 2) and BT-549 (mesenchymal), which expressed high levels of MELK and were used for knockdown or KO experiments (Fig. 2A; top right); and SUM149 (basal-like 2) and SUM159 (mesenchymal stem-like), which expressed low levels of MELK and were used for overexpression experiments (Fig. 2B; top). Compared with SCR control, MELK knockdown by siMELK #1 or siMELK #5 reduced cell proliferation by 23% (*P* < 0.01) and 45% (*P* < 0.0001), respectively, in HCC70 cells; and by 24% (*P* < 0.05) and 77% (*P* < 0.0001), respectively, in BT-549 cells (Fig. 2A). In agreement with these findings, MDA-MB-231 MELK KO clones showed reduced anchorage-independent growth by 55% in C3 clones (*P* < 0.05) and 71% in C28 clones (*P* < 0.05; Fig. 2A, right) compared with Cas9-p15 control cells. To further confirm the requirement of MELK kinase activity for TNBC cell growth, we assessed the effects of WT or KD MELK overexpression on TNBC cell growth using the clonogenic assay. Overexpression of WT MELK increased colony formation by 36.76% compared with vector (*P* < 0.01) and by 45.76% compared with KD MELK (*P* < 0.01) in SUM149 cells, and by 30.03% compared with vector (*P* < 0.01) and by 73.99% compared with KD MELK (*P* < 0.0001) in SUM159 cells (Fig. 2B). These results suggest that MELK promotes TNBC cell growth and that MELK kinase activity is required.

Because high MELK expression correlates with metastasis in patients with breast cancer (17) and because the function of MELK in TNBC metastasis has not been investigated, we examined the effects of MELK knockdown on migration and invasion, the two key early processes of metastasis (35). Compared with SCR control, MELK knockdown using siRNA targeting two different sequences significantly impaired migration and invasion of both HCC70 and BT-549 cells (Fig. 2C). MELK knockdown in HCC70 cells using siMELK #1 and siMELK #5 reduced migration by 49% (*P* < 0.001) and 75% (*P* < 0.0001), respectively, and invasion by 52% (*P* < 0.001) and 70% (*P* < 0.001), respectively. MELK knockdown in BT-549 cells using siMELK #1 and siMELK #5 reduced migration by 38% (*P* < 0.001) and 79% (*P* < 0.0001), respectively, and invasion by 42% (*P* < 0.05) and 49% (*P* < 0.05), respectively. To further confirm the requirement of MELK kinase activity for TNBC cell invasiveness, we assessed the effects of WT or KD MELK overexpression on TNBC cell migration and invasion. Overexpression of WT MELK increased migration by 102.17% compared with vector (*P* < 0.0001) and by 141.15% compared with KD MELK (*P* < 0.0001) and increased invasion by 42.47% compared with vector (*P* < 0.001) and by 60.73% compared with KD MELK (*P* < 0.0001) in SUM149 cells (Fig. 2D). Similarly, overexpression of WT MELK increased migration by 60.12% compared with vector (*P* < 0.0001) and by 97.68% compared with KD MELK (*P* < 0.0001) and increased invasion by 33.58% compared with vector (*P* < 0.01) and by 94.74% compared with KD MELK (*P* < 0.0001) in SUM159 cells (Fig. 2D). These results suggest that MELK promotes invasiveness of TNBC cells and that MELK kinase activity is required.

### MELK Induces EMT and a CSC Phenotype in TNBC

Our findings suggest that MELK contributes to the invasiveness and migratory traits of TNBC cells. Therefore, we wanted to further investigate the potential link between MELK and EMT and CSCs. Previous research has shown that EMT and the CSC phenotype are important in metastasis (36). We examined the contribution of MELK to the induction of EMT in breast cancer cells by determining the impact of MELK knockdown or inhibition on the expression of epithelial and mesenchymal markers using Western blot analysis. We found that MELK knockdown reduced the expression of mesenchymal markers fibronectin and snail in HCC70 cells (Fig. 2E).

Because EMT is known to induce the generation of breast cancer cells with stem cell–like characteristics (6), we next determined whether MELK is capable of inducing a CSC phenotype in TNBC by examining the effects of MELK knockdown or overexpression on mammosphere formation (37) and CSC phenotype (CD44^+^/CD24^−^ and ALDH1^+^ subpopulations) using flow cytometry. Compared with SCR control, MELK knockdown using siMELK #1 or siMELK #5 reduced the formation of mammospheres (which are enriched for stem cells) by 37% (*P* < 0.001) and 34% (*P* < 0.01), respectively, in HCC70 cells and by 31% (*P* < 0.001) and 39% (*P* < 0.001), respectively, in BT-549 cells (Fig. 2F). Consistent with these findings, in MDA-MB-231 cells, MELK KO decreased the formation of mammospheres by 28% in C3 clones (*P* < 0.0001) and by 29% in C28 clones (*P* < 0.001; Fig. 2F) compared with Cas9-p15 control cells. To further confirm the requirement of MELK kinase activity for self-renewal of TNBC CSCs, we assessed the effects of WT or KD MELK overexpression on mammosphere formation. Overexpression of WT MELK increased mammosphere formation by 72.99% compared with vector (*P* < 0.001) and by 80.67% compared with KD MELK (*P* < 0.0001) in SUM149 cells and by 57.68% compared with vector (*P* < 0.001) and by 75.78% compared with KD MELK (*P* < 0.0001) in SUM159 cells (Fig. 2G). Compared with SCR control, MELK knockdown in BT-549 cells also reduced the proportion of cells with CD44^+^/CD24^−^ surface markers (1.9% by siMELK #1 and 5.9% by siMELK #5) and the proportion of cells with ALDH1 activity (4.6% by siMELK #1 and 42% by siMELK #5; Fig. 2H).

These results suggest that MELK induces EMT and at least partially promotes self-renewal and maintenance of CSCs in TNBC.

### MELK-in-17 Suppresses Proliferation, Motility, and CSC Self-renewal and Reverses EMT in TNBC Cells *in Vitro*

**FIGURE 3:**
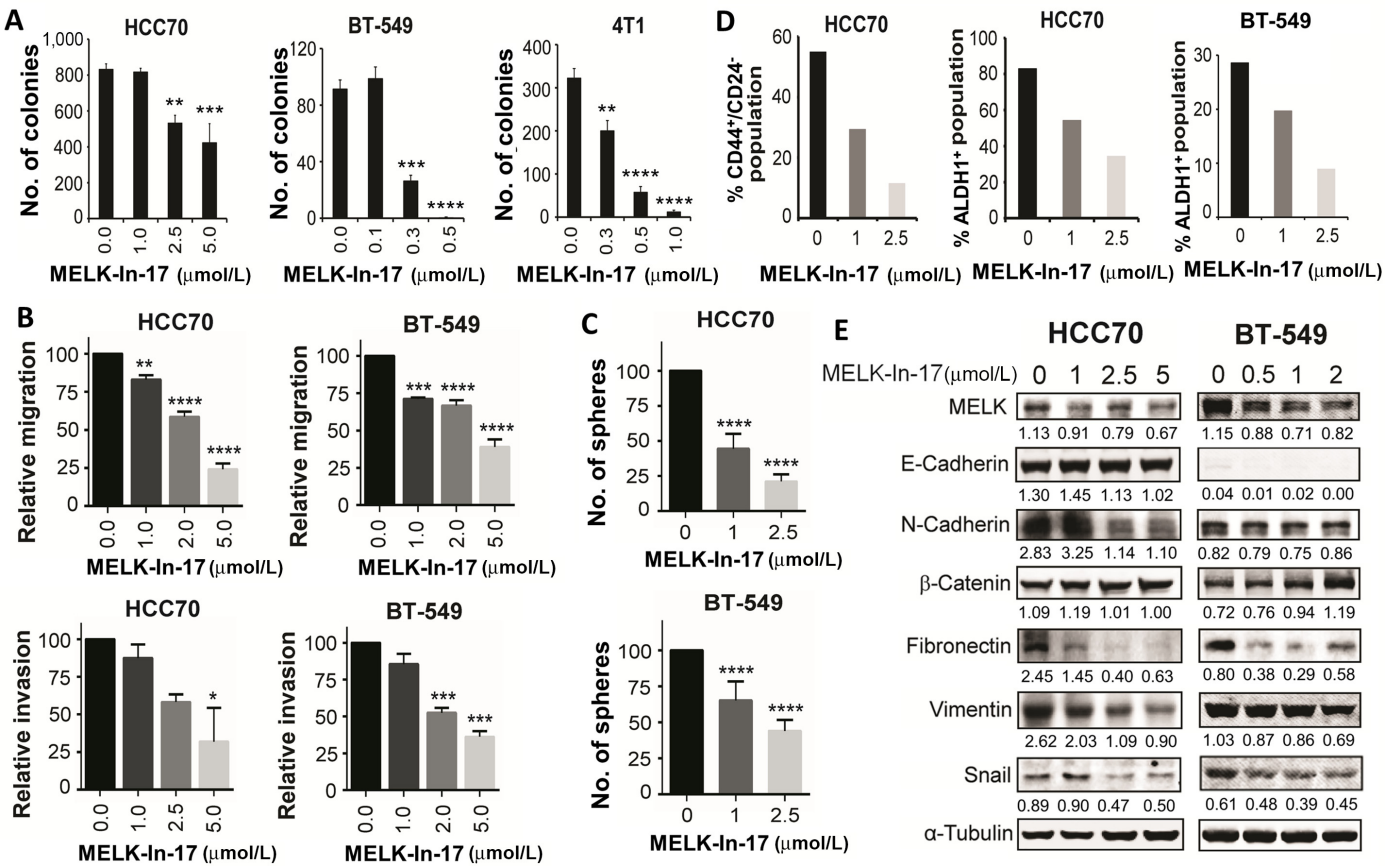
Inhibitory effect of MELK-In-17 on anchorage-independent growth, motility, CSC properties, and EMT transition in TNBC cells. **A–D,** MELK-In-17 inhibited anchorage-independent growth (**A**), suppressed migration and invasion (**B**), reduced mammosphere formation (**C**), and reduced CD44^+^/CD24^−^ and ALDH1^+^ CSC subpopulations (**D**) in TNBC cells. In A, the anchorage-independent growth was determined using a soft-agar assay at week 3 following treatment. In B, cells were pretreated with MELK-In-17 for 2 hours and then assayed for migration and invasion in the presence of MELK-In-17. In C, cells were seeded for mammosphere formation and the next day treated with MELK-In-17. In D, cells were treated with MELK-In-17 for 48 hours and then subjected to flow cytometry analysis. **E,** Western blot analysis of the effects of treatment with MELK-In-17 on the expression of EMT markers. α-Tubulin was used as a loading control. Band intensity of proteins is normalized to that of α-Tubulin. In A–C, data are presented as mean ± SD. *, *P* < 0.05; **, *P* < 0.01; ***, *P* < 0.001; ****, *P* < 0.0001.

To confirm our findings that MELK promoted cell growth, migration, and invasion and induced EMT and the CSC phenotype in TNBC, we further examined whether MELK inhibition by MELK-In-17 would have the same effects as MELK knockdown did. MELK-In-17 possesses an indolinone scaffold and is a highly potent MELK inhibitor (26). MELK-In-17 exhibits good selectivity toward MELK over MELK's most closely related family members AMPK and NUAK1 by 24- and 28-fold, respectively (26). MELK-In-17 forms hydrogen bonds with C89 (2.7 Å), E87 (2.9 Å), and K40 (3.7 Å) of the ATP binding pocket of MELK, thereby inhibiting the catalytic domain of MELK (26). Our study showed that MELK-In-17 significantly reduced anchorage-independent growth (an indicator of *in vivo* tumorigenicity) of HCC70, BT-549, and 4T1 cells in a dose-dependent manner (Fig. 3A). This result further suggests that MELK regulates TNBC cell growth.

We next examined the effects of MELK-In-17 on cell motility, EMT, and the CSC phenotype in TNBC cells. As shown in Fig. 3B, MELK-In-17 significantly suppressed migration and invasion of HCC70 and BT-549 cells in a dose-dependent manner. Furthermore, MELK-In-17 significantly inhibited mammosphere formation (Fig. 3C) and CD44^+^/CD24^−^ and ALDH1^+^ CSC subpopulations (Fig. 3D) in HCC70 and BT-549 cells, suggesting that MELK plays a role in the promotion of the CSC phenotype in TNBC. Moreover, MELK-In-17 suppressed the expression of mesenchymal markers (E-cadherin, N-cadherin, fibronectin, vimentin, or snail) and increased the expression of β-catenin in HCC70 and BT-549 cells (Fig. 3E). MELK-In-17 also suppressed the expression of mesenchymal markers (E-cadherin, N-cadherin, fibronectin, vimentin, and snail) at mRNA levels in 4T1 cells (Supplementary Fig. S1). These data suggest that that MELK is essential for EMT in TNBC.

Taken together, these results suggest that MELK is essential for the promotion of proliferation, motility, EMT, and the CSC phenotype in TNBC.

### MELK Promotes Tumorigenesis and Metastasis in TNBC Xenograft Models

**FIGURE 4:**
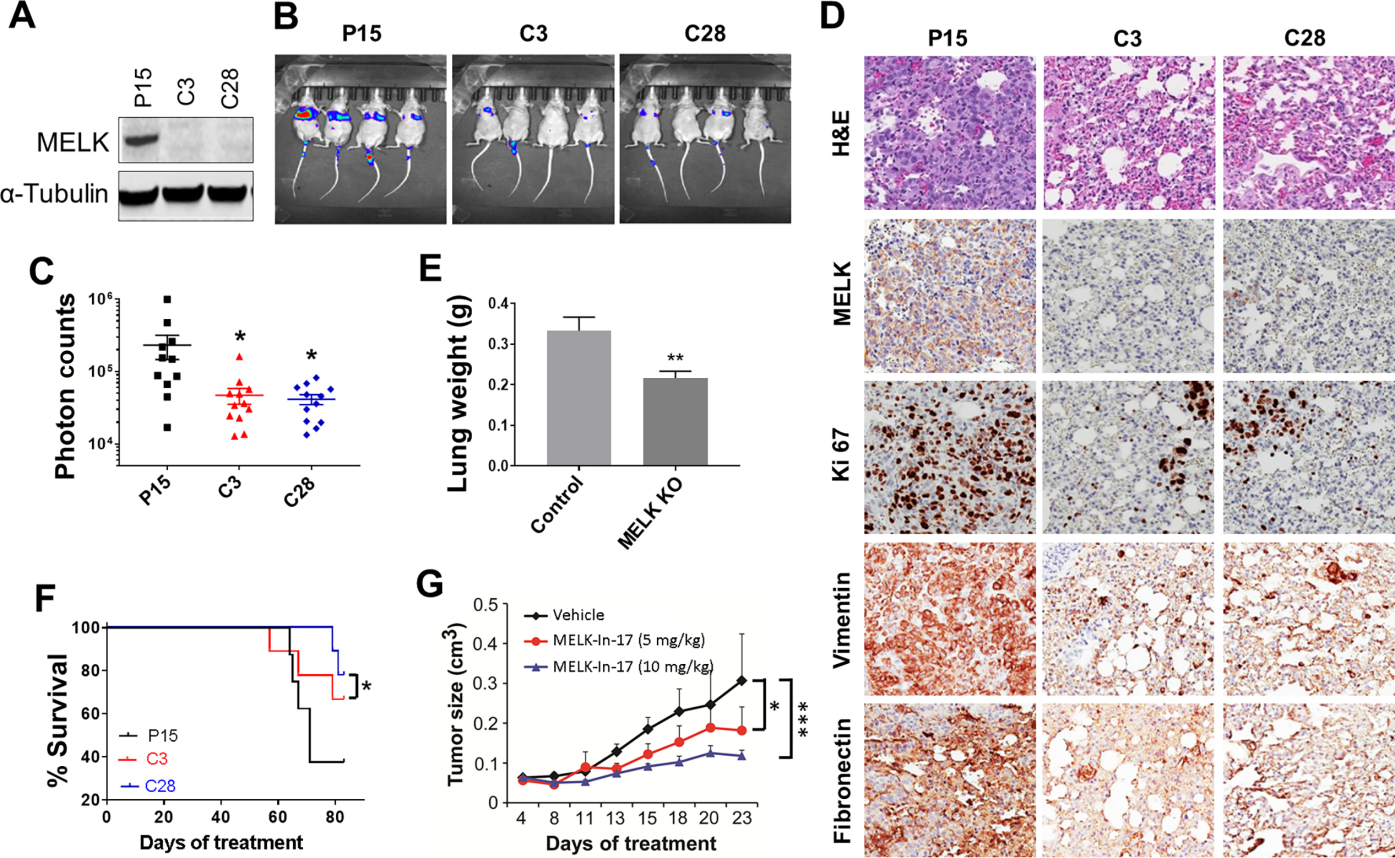
Effect of inhibition of MELK signaling on tumor growth and lung metastasis in TNBC xenograft mouse models. **A,** MELK expression in Cas9-p15 (P15) and MELK KO MDA-MB-231 (C3 and C28) cells. α-Tubulin was used as a loading control. **B–F,** MELK KO significantly suppressed lung metastases in an MDA-MB-231 xenograft mouse model. Female athymic nude mice were injected intravenously with luciferase-transfected Cas9-p15 or MELK KO MDA-MB-231-Luc-GFP stable cells. Metastatic tumors were measured weekly for 7 weeks by whole-body luciferase imaging using an IVIS 100 Imaging System. Shown are mouse whole-body luciferase images (**B**), photon counts per area (**C**), lung weight per mouse measured at week 7 following cell inoculation (**D**), mouse OS over a period of 80 days following cell inoculation (**E**), and IHC staining for hematoxylin and eosin (H&E), MELK, proliferation (Ki67), and mesenchymal markers (vimentin and fibronectin; **F**) in mice inoculated with Cas9-p15 or MELK KO MDA-MB-231 cells. Images were taken under 200 × magnification. **G,** MELK-In-17 significantly suppressed tumor growth in a 4T1 xenograft mouse model (*P* < 0.05). Murine 4T1 TNBC cells were injected into the mammary fat pads of female BALB/c mice. When tumor size reached 75–150 mm^3^, grouped mice were treated with vehicle or MELK-In-17 at 5 or 10 mg/kg via intraperitoneal injections daily for 25 days. In C–G, data are presented as mean ± SD. *, *P* < 0.05; **, *P* < 0.01.

Upon observing that MELK KO cells had reduced aggressiveness *in vitro*, we next tested whether loss of MELK inhibited the metastasis of TNBC cells *in vivo*. Control Cas9-p15 and MELK KO MDA-MB-231 (C3 and C28; Fig. 4A) cells labeled with the Luc-GFP fusion protein were injected into the tail vein of nude mice to examine lung metastasis *in vivo*. The bioluminescent signal was examined weekly to monitor metastatic sites and the growth of tumors. At week 6, we found that the photon flux, which represents pulmonary metastasis foci, was significantly higher in control Cas9-p15 cells than in MELK KO cells (*P* < 0.05; Fig. 4B and C), and this was also evident in hematoxylin and eosin staining (Fig. 4D). The lungs of mice injected with the control cells weighed significantly more than those injected with MELK KO cells (*P* < 0.01; Fig. 4E).

Furthermore, suppression of lung metastasis by MELK KO significantly improved OS compared with the control mice (*P* < 0.05; Fig. 4F). IHC staining showed that the control lung tissues displayed metastatic poorly differentiated carcinoma distributed as nodules ranging from <1 to 1.5 mm in maximum dimension. Hematoxylin and eosin staining showed that control tissues contained approximately 95% tumor cells, whereas the number of nodules was lower in MELK KO tissues. There was also a marked reduction in the size of metastatic tumor nodules. IHC staining confirmed MELK KO and showed a reduction in the expression of Ki67, vimentin, and fibronectin in tumors from mice inoculated with MELK KO cells (Fig. 4D), suggesting that MELK KO reduces proliferation of tumor cells and inhibits EMT *in vivo* and thereby suppresses lung metastasis. Our data collectively indicate that MELK KO MDA-MB-231 cells showed a profound decrease in the metastatic potential to the lungs, thus resulting in a substantial extension of survival.

We next tested whether MELK inhibition by MELK-In-17 suppresses TNBC tumorigenesis *in vivo*. We treated BALC/c mice bearing murine 4T1 TNBC tumors with MELK-In-17 and examined the effect on tumor growth. The inhibitor significantly suppressed tumor growth after daily injections in a dose-dependent manner (compared with the control vehicle, *P* < 0.001; Fig. 4G). These results suggest that MELK plays an important role in TNBC tumorigenesis.

### MELK is Associated with Expression of Genes Associated with EMT, Tumor Progression, and Metastasis

**FIGURE 5:**
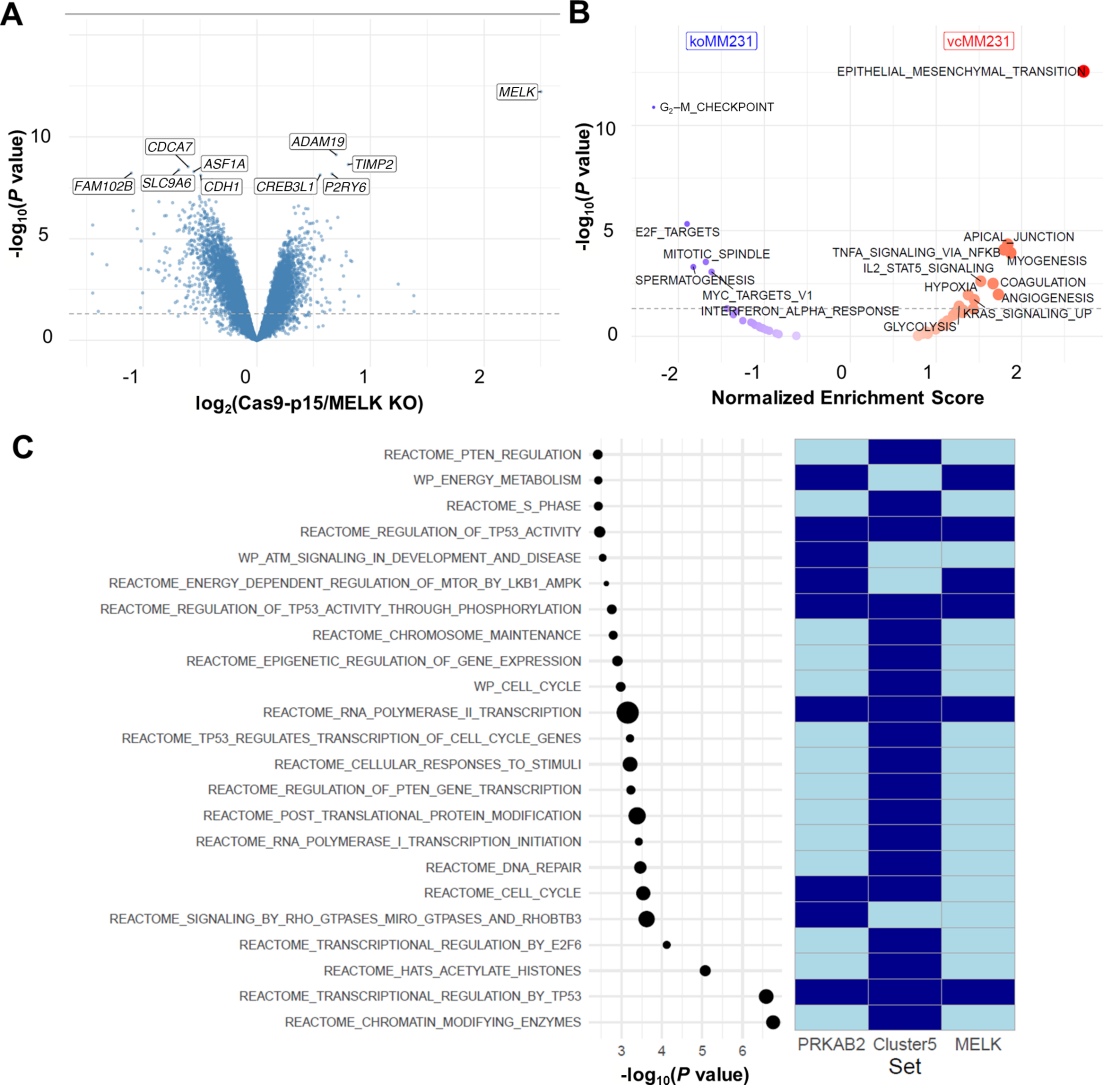
Differential gene expression profile, consolidated gene set enrichment, and network analyses. **A,** Differences in gene expression are shown in volcano plot format, where the *X*-axis denotes the log_2_-transformed fold-change and the *Y*-axis denotes the –log_10_-transformed *P* value. The log_2_ fold-change was calculated as Cas9-p15 control cell fold-change/MELK KO MDA-MB-231 cell fold-change, resulting in positive values for genes overexpressed in the WT condition and negative values for genes overexpressed in the KO condition. The nominal *P* value threshold at 5% is indicated by a dashed blue horizontal line. The 10 most differentially expressed genes by *P* value are labeled using their gene symbol. **B,** Differences in the hallmark enrichment pattern are shown in a modified volcano plot format, where the *X*-axis denotes the normalized enrichment score and the *Y*-axis denotes the –log_10_-transformed *P* value. The normalized enrichment score was calculated as Cas9-p15 control cell score/MELK KO MDA-MB-231 cell score, resulting in positive values for hallmarks enriched in the WT condition and negative values for hallmarks enriched in the KO condition, as indicated at the top of the plot. The nominal *P* value threshold at 5% is indicated by a dashed blue horizontal line. Significant hallmarks are labeled using their gene set name. **C,** Pathway enrichment profile of the MELK-centered PPI network. In the left part of the plot, significantly enriched pathways, listed along the *Y*-axis, are represented in dot plot format; the *X*-axis represents the –log_10_-transformed *P* value; and the size of the dots reflects the number of pathway genes contained in the network. To the right of the dot plot, overlaps between enriched pathways and three gene sets defined after analyzing the MELK-centered protein–protein interaction network are indicated in heat map format. The gene sets of interest are indicated in the *X*-axis underneath the heat map, where dark blue denotes the presence of pathway genes and light blue denotes the absence of pathway genes in these gene sets.

A comparison of gene expression profiles between Cas9-p15 control and MELK KO MDA-MB-231 cells was performed to explore the biological implications of MELK KO in TNBC. Differential gene expression profile analysis identified 2,537 upregulated and 2,819 downregulated genes in MELK KO MDA-MB-231 cells compared with Cas9-p15 control cells, at a FDR of 10%. MELK was the top differentially expressed gene, with high levels of expression in Cas9-p15 control cells relative to MELK KO cells (fold-change of 5.663). The corresponding volcano plot depicting gene expression differences between Cas9-p15 control and MELK KO MDA-MB-231 cells is shown in Fig. 5A. The remaining volcano plots are shown in Supplementary Fig. S2.

Vectors of log_2_-transformed fold-changes between Cas9-p15 control and MELK KO MDA-MB-231 cells were translated into biological themes using gene set enrichment analysis, identifying EMT as the most strongly enriched hallmark among genes highly expressed in Cas9-p15 control cells. In contrast, the hallmark G_2_–M checkpoint was the top enriched concept among genes highly expressed in MELK KO cells. In general, genes highly expressed in MELK KO cells showed enrichment for gene sets associated with cell proliferation (i.e., G_2_–M checkpoint genes, E2F target genes, MYC target genes, and mitotic spindle genes), and high expression of MELK-induced high levels of expression of *STAT5* and *NFKB* target genes, as well as genes associated with concepts generally involved in tumor progression and metastasis (i.e., angiogenesis, hypoxia, coagulation, and apical junction). Results are shown in a modified volcano plot in Fig. 5B.

### MELK Induces Pathway Deregulation

To identify candidate regulators that contribute to the MELK-induced gene expression changes, and thus underpin the above-described biological expression themes, we performed VIPER analysis to identify expression differences between Cas9-p15 control and MELK KO MDA-MB-231 cells. After correction for pleotropic effects, 1,365 proteins with a differential activity profile were identified. Enrichment plots for the top 20 candidate regulators are shown in Supplementary Fig. S3. The full list of candidate regulators was significantly enriched for 60 Reactome and WikiPathways, which are provided in Supplementary Table S1.

Next, using the protein–protein interaction (PPI) network STRING, we calculated all shortest paths between MELK and any of the candidate regulators identified by VIPER. In total, 2,446 shortest paths were identified, with a length ranging from 2 to 7. On the basis of this analysis, only one candidate regulator (PRKAB2) was identified as a direct physical interaction partner of MELK. In addition, 111 of 1,365 candidate regulators interacted with MELK through at most one intermediate protein. The set of intermediate proteins was limited to CDC25B, EZH2, FOXM1, JUN, MAP3K5, PRKAB1, PRKAB2, and SMAD2, suggesting that these proteins are key components of the MELK-induced signal transduction mechanisms. The PPI network restricted to the 112 candidate regulators (i.e., PRKAB2 and 111 candidate regulators with indirect MELK interactions) identified in this analysis, as well as their intermediate proteins, is shown in Supplementary Fig. S4A.

Next, based on the resulting PPI network, two prioritization strategies were applied, and results were used to filter the set of 60 significantly enriched Reactome and WikiPathways identified earlier. Results are shown in Fig. 5C. First, because PRKAB2 directly interacts with MELK and also serves as an intermediate protein to connect MELK with four other candidate regulators (i.e., BLZF1, MDFI, STK11, and TRAK2), pathways containing the PRKAB2 connectome were retained. Second, within the PPI network, five clusters were identified (Supplementary Fig. S4B). Of these, the fifth cluster contained 17 candidate regulators with low VIPER activity scores following MELK activation (i.e., ASXL2, ASXL3, ATRX, CDK2, CRY2, E2F4, E2F5, E2F6, KDM5A, LCOR, LIN54, MTF2, PHF1, RB1, RBBP7, RBL1, and SOX4), whereas activity scores in the remaining clusters were not significantly different from zero (Supplementary Fig. S4C). Given this obvious trend, pathways containing any of the candidate regulators contained in cluster 5 were also retained. For all 23 retained pathways, overlap with the list of eight intermediate proteins was also evaluated. Together, these results indicate that MELK regulates the cell cycle, energy metabolism, DNA and RNA metabolism, transcription, and PTEN and TP53 signal transduction pathways in TNBC.

## Discussion

MELK is a multifunctional protein implicated in tumor progression in several cancers (17–20). Our gene expression profile studies showed that *MELK* mRNA expression was highly elevated in TNBC and that high *MELK* mRNA expression was associated with a high risk of death in patients with breast cancer. MELK also played a key role in TNBC cell invasiveness, CSC maintenance, and EMT induction. More importantly, MELK inhibition with a novel selective MELK inhibitor suppressed tumor growth, and MELK KO suppressed lung metastasis in TNBC xenograft mouse models. Our findings indicate that MELK promotes a CSC phenotype, EMT induction, and metastasis in TNBC.

MELK has been considered a prognostic factor to predict breast tumor recurrence. Consistent with findings reported by Wang and colleagues showing that MELK is most highly overexpressed in TNBC (17), our analysis of tumor tissues from 314 patients with breast cancer showed that *MELK* mRNA expression was significantly higher in patients with TNBC than in patients with the HR^+^HER2^−^ subtype. Furthermore, in a univariate analysis, patients with breast cancer with tumors expressing high mRNA levels of *MELK* had significantly reduced PFS, DMFS, and OS compared with patients with tumors expressing low levels of *MELK*. However, in a multicovariate analysis, high *MELK* mRNA expression was associated with shorter OS but did not have independent prognostic value for PFS or DMFS. Recent studies also showed that MELK is overexpressed in other types of cancers, such as brain cancer, colon cancer, glioblastoma, and melanoma (17, 38–40), and high levels of MELK are correlated with tumor grade, poor prognosis, radioresistance, and recurrence in multiple cancers (19, 21, 41–45). These findings indicate that MELK is a promising therapeutic target for multiple cancers.

MELK plays critical roles in various cellular and biological processes, including the cell cycle, proliferation, apoptosis, spliceosome assembly, gene expression, embryonic development, and hematopoiesis (16). In addition, MELK is involved in tumorigenic processes; MELK knockdown was shown to reduce tumorigenic properties in multiple tumor models (15, 21, 43). In agreement with these studies, our results showed that abrogation of MELK expression by siRNA or CRISPR/Cas9 or inhibition of MELK activity by MELK-In-17 suppressed proliferation and invasiveness of TNBC cells, whereas overexpression of MELK had the opposite effect. More importantly, MELK KO and treatment with MELK-In-17 suppressed lung metastasis and tumor growth in animal models. MELK is also known to regulate CSC self-renewal, and MELK expression is elevated in CSCs (16), suggesting that dysregulation of MELK may cause carcinogenesis in various cell types. In support of this notion, our study showed that knockdown of MELK expression or inhibition of MELK activity reduced CSC properties and reversed EMT in TNBC. The CSC phenotype and the EMT process are essential for tumor initiation and metastasis. Therefore, MELK might promote tumorigenesis in TNBC through its effects on CSCs and EMT.

MELK has previously been implicated as an important factor in various cancers. However, the role of MELK in the regulation of cell proliferation has been controversial. MELK knockdown by siRNA or MELK inhibition by inhibitors showed a strong growth-inhibitory effect on cancer cells (40). In contrast, MELK KO by CRISPR/Cas9 showed conditional or no effect on the proliferation of cancer cells (46–48). Studies by Giuliano and colleagues and Lin and colleagues showed that MELK deletion by CRISPR/Cas9 in CAL-51 and MDA-MB-231 TNBC cells had little effect on the proliferation of these cancer cells (46, 49). MELK KO clones grew normally in cell culture and xenograft mouse models. In addition, multiple high-throughput genetic screens in multiple cell lines, including TNBC cell lines, did not identify MELK as a potential cancer target (46). In contrast, we found that MELK depletion using siRNA significantly suppressed proliferation, migration, and invasion in high-MELK–expressing HCC70 and BT-549 cells, whereas overexpression of MELK in low-MELK–expressing SUM149 and SUM159 cells had the opposite effect. However, MELK KO in MDA-MB-231 cells by CRISPR/Cas9 had no effect on cell proliferation in two-dimensional culture but reduced anchorage-independent growth, suggesting that the effect may be related to the microenvironment and growth conditions.

In our xenograft mouse model, MELK KO in MDA-MB-231 cells also suppressed lung metastasis. Wang and colleagues suggested that disparities in these findings may originate from fundamental differences in the target validation methods rather than the choice of genetic tools (47). Wang and colleagues showed that subtle technical variation leads to dramatic differences in experimental outcomes (47). For instance, MELK knockdown by RNA inhibition or CRISPR/Cas9 reduced clonogenic growth of TNBC cells. In contrast, MELK abrogation had no appreciable effects on cell proliferation under the common “short-term, high-density” culture conditions. Furthermore, McDonald and colleagues suggested that the complete loss of protein expression and activity by CRISPR/Cas9 could have different effects than partial inhibition of protein expression and activity by RNA inhibition of specific inhibitors (50). Genomic KO could lead to cellular reprogramming of signaling networks to compensate for growth defects caused by specific protein KO. In contrast, acute partial inhibition or depletion of the same protein may not result in the same changes. At this point, these potential explanations are purely speculative, but this will undoubtedly be an important area of investigation for future MELK studies. The findings of Wang and colleagues highlight the importance of experimental design and technical considerations in cancer target validation and reconcile some of the disparities in the current literature regarding MELK dependency in cancer progression.

Owing to advances in studies of oncogenic signal transduction pathways, targeted therapies have made great progress in cancer treatment. Inhibitors targeting highly conserved regions of kinases are generally not highly selective in their targets, and the efficacy of these targeted therapies relies on the abundance of the target relative to other affected kinases. Although several molecules have been reported to inhibit MELK (18, 51, 52), no potent MELK inhibitors with demonstrated selectivity have been reported. For instance, OTS167 is highly effective in preclinical studies and is currently in a phase I study; however, OTS167 cross-reacts with many essential kinases and decreases the activity of up to 210 kinases when applied at a high concentration (10 μmol/L; ref. 53), which can cause unwanted side effects (54). Therefore, to leverage MELK's role in breast cancer metastasis as a therapeutic target, we need to identify specific and potent MELK inhibitors. Previously, we discovered a highly selective MELK inhibitor, MELK-In-17, using an off-target/cross-screening assay of an in-house library of approximately 800 known kinase inhibitors (26). The antiproliferation effect of MELK-In-17 largely depended on expression levels of MELK in TNBC cells (26), suggesting high selectivity. In the current study, we also showed that MELK-In-17 suppressed the CSC phenotype and reversed EMT *in vitro* and inhibited tumor growth in mouse models, further supported by the impaired tumor growth in mice bearing xenografts of MDA-MB-231 cells in which MELK was knocked out. These studies indicate the therapeutic potential of MELK-In-17 for the treatment of TNBC and other cancers.

The role of MELK in cancer has been of increasing interest because of its elevated expression in various cancer tissues and its association with poor patient outcomes. Our findings demonstrate the clinical significance of MELK in TNBC and the importance of MELK in the promotion of proliferation and invasiveness of TNBC cells and CSC self-renewal. In our microarray analysis, we identified potential downstream targets of MELK, including *STAT5* and *NFKB* target genes, as well as genes involved in tumor progression and metastasis (i.e., EMT, angiogenesis, hypoxia, and apical junction). EMT was the most strongly enriched hallmark among genes highly expressed in Cas9-p15 control cells, further confirming that EMT is a major factor contributing to MELK-induced metastasis in TNBC. We also identified a direct physical interaction partner (PRKAB2) of MELK and a set of intermediate proteins (CDC25B, EZH2, FOXM1, JUN, MAP3K5, PRKAB1, PRKAB2, and SMAD2), suggesting that these proteins are key components of MELK-induced signal transduction. We are currently exploring these molecules to uncover the molecular mechanism involved. High MELK expression has been associated with immune cell infiltration and pathologic complete response after neoadjuvant chemotherapy in breast cancer (55). Therefore, there remains a need to determine the role of MELK in modulating the tumor microenvironment, which is a critical component of breast cancer response to treatment.

## Supplementary Material

Supplementary Materials and Methods Supplementary Materials and Methods

Supplementary Figure S1 Figure S1. Inhibitory effect of treatment with MELK-In-17 on the expression of epithelial-to-mesenchymal transition markers in murine 4T1 triple-negative breast cancer cells.

Supplementary Figure S2 Supplementary Figure S2. Volcano plots show differences in gene expression in parental and Cas9-p15 control or MELK knockout (KO) MDA-MB-231 cells.

Supplementary Figure S3 Supplementary Figure S3. Top 20 master regulators that were differentially expressed in Cas9-p15 control and MELK knockout (KO) MDA-NB-231 cells.

Supplementary Figure S4 Supplementary Figure S4. Graphical representation of the physical protein-protein interaction network involved in modulating MELK activity in MDA-MB-231 cells.

Supplementary Table S1 Supplementary Table S1. Full list of candidate regulators that were significantly enriched for 60 Reactome and WikiPathways, as identified by Affymetrix Genechip microarray analysis.
