# Correction: Maternal Embryonic Leucine Zipper Kinase Is Associated with Metastasis in Triple-negative Breast Cancer

**DOI:** 10.1158/2767-9764.CRC-24-0046

**Published:** 2024-01-29

**Authors:** Xuemei Xie, Gaurav B. Chauhan, Ramakrishna Edupuganti, Takahiro Kogawa, Jihyun Park, Moises Tacam, Alex W. Tan, Mohd Mughees, Fnu Vidhu, Diane D. Liu, Juliana M. Taliaferro, Mary Kathryn Pitner, Luke S. Browning, Ju-Hyeon Lee, François Bertucci, Yu Shen, Jian Wang, Naoto T. Ueno, Savitri Krishnamurthy, Gabriel N. Hortobagyi, Debu Tripathy, Steven J. Van Laere, Geoffrey Bartholomeusz, Kevin N. Dalby, Chandra Bartholomeusz

In the original version of this article (1), author François Bertucci is mistakenly omitted from the author list. This error has been corrected in the latest online HTML and PDF versions of the article and the author contributions and disclosures have been updated as necessary. The authors regret this error.
