# Supplementary material for: Maternal Embryonic Leucine Zipper Kinase is Associated with Metastasis in Triple-negative Breast Cancer
Source: Cancer Res Commun. 2023 Jun 20;3(6):1078–92. doi: 10.1158/2767-9764.CRC-22-0330 (PMC10281291; doi:10.1158/2767-9764.CRC-22-0330)
Supplement: Supplementary Materials and Methods [file crc-22-0330-s01.docx]

**Supplementary Materials and Methods**

**Cell proliferation assay**

The trypan blue exclusion assay was performed to determine the effects of MELK knockdown on cell proliferation (1). HCC70 and BT-549 cells (2 × 10^5^ cells/well) were seeded in 6-well plates and transfected the next day with scrambled siRNA or siMELK. Seventy-two hours later, cells were harvested and stained with trypan blue, and dead cells were counted using a hemocytometer under a microscope. The anti-proliferation efficacy of MELK-In-17 was determined using the CellTiter-Blue Viability Assay (2). Cells were seeded in 96-well plates and treated the next day with MELK-In-17 (0-20 µM). At 72 hours after treatment, optical density at 595 nm was determined.

**Clonogenic assay**

The clonogenic assay was performed to determine the effect of MELK overexpression on cell growth (3). SUM149 and SUM159 cells were transfected with the pCMV6-AC-GFP empty vector (OriGene, Rockville, MD, USA) or the pCMV6-AC-GFP-MELK vector carrying wild-type (WT) MELK (OriGene) or kinase-dead (KD) MELK using the Neon Transfection System (Life Technologies Inc, Grand Island, NY, USA) according to the manufacturer’s instructions. At 24 hours after transfection, SUM149 and SUM159 cells (500 cells/well) were seeded in 6-well plates and then incubated at 37 °C for 5-7 days. After incubation, the colonies were stained with MTT (3-[4,5-dimethylthiazol-2-yl]-2,5-diphenyltetrazolium bromide; Thermo Fisher Scientific, Waltham, MA, USA), and colonies >80 µm in diameter were counted using the GelCount system (Oxford Optronix Ltd, Milton Park, Abingdon, UK).

**Anchorage-independent growth evaluation**

To assess anchorage-independent growth, an indicator of *in vivo* tumorigenicity (4), we suspended cells in 0.5% agarose in complete medium containing MELK-In-17 at the indicated doses. The mixture was overplayed on a layer of 1% agarose in complete medium in 6-well plates. The top layer was allowed to solidify at 4 °C for 15 minutes, and the plates were then incubated at 37 °C for 3 weeks. After 3 weeks of treatment, the colonies were stained with MTT, and colonies >80 µm in diameter were counted using the GelCount system (Oxford Optronix Ltd). To determine the effect of MELK knockout (KO) on anchorage-independent growth of TNBC cells, we suspended MDA-MB-231 MELK KO cells and Cas9-p15 control cells in 0.5% agarose in complete medium. The cell suspension was then overlayed on a layer of 1% agarose in complete medium in 6-well plates and incubated for 3 weeks. After a 3-week incubation, colonies >80 µm in diameter were counted using the GelCount system.

**Cell migration and invasion assays**

Migration assays were performed using a 24-well microchemotaxis chamber (Corning Inc, Corning, NY, USA). After transfection with siRNA or vector carrying the coding sequences for WT MELK or KD MELK, cells were resuspended in fetal bovine serum (FBS)-free medium (2.5 × 10^5^ cells/250 µL) and then added into the upper chambers of transwells separated by inserts with 8-µm pores. The lower chambers were filled with complete medium (750 µL) containing 10% FBS as an attractant. The cells were allowed to migrate for 6-8 hours and then fixed and stained with hematoxylin and eosin. Invasion assays were performed using a 24-well microchemotaxis chamber as described above for migration assays, with the following modifications. The upper chambers of transwells were pre-coated with 100 µL of Matrigel diluted at 1:15 in FBS-free medium, and cells were allowed to invade for 24 hours. Migrated or invaded cells were scanned using the PathScan Enabler IV Histology Slide Scanner (Meyer Instruments, Inc, TX, USA) and then quantified using National Institutes of Health Image J software (<http://rsb.info.nih.gov/ij/>). To determine the effects of treatment with MELK-In-17 on migration and invasion of TNBC cells, we pre-treated cells with MELK-In-17 for 2 hours and then assayed for migration and invasion as described above in the presence of MELK-In-17 at 0, 1, 2, and 5 µM.

**Mammosphere assay**

To determine the effects of MELK knockdown or overexpression on mammosphere formation, at 48 hours after transfection with siRNA or vector carrying coding sequences for WT MELK or KD MELK, we prepared single-cell suspensions in TeSR-E8 medium (STEMCELL Technologies Inc, Vancouver, BC, Canada) supplemented with mammoCult proliferation supplements, 1% antibiotic/antimycotic, 4 µg/mL heparin, and 0.48 µg/mL hydrocortisone. The cell suspensions (2 × 10^4^ cells in 2 mL) were then seeded in 6-well ultra-low attachment plates (Corning Inc, Corning, NY, USA) and incubated for 7 days. After 7-day incubation, mammospheres were stained with MTT, and mammospheres >80 µm in diameter were counted using the GelCount system (Oxford Optronix Ltd). To determine the effect of MELK KO on mammosphere formation, we cultured 2 × 10^4^ MDA-MB-231 MELK KO cells and Cas9-p15 control cells in 2 mL of TeSR-E8 medium for 7 days, followed by mammosphere measurement as described above. To determine the effect of MELK inhibition on mammosphere formation, we cultured 2 × 10^4^ cells in 2 mL of TeSR-E8 medium with or without MELK-In-17 at 0, 1, and 2.5 µM for 7 days. On day 7 following treatment, mammospheres were counted as described above.

**Cancer stem-like cell subpopulation analysis**

To determine the effect of MELK knockdown on cancer stem-like cell subpopulations, at 72 hours after treatment with scrambled siRNA or siMELK, we harvested cells and incubated them at 37 °C with ALDEFLUOR reagent (STEMCELL Technologies Inc) or at room temperature with anti-CD24 and anti-CD44 antibodies (BD Biosciences, San Jose, CA, USA) for 30 minutes and then subjected them to flow cytometry analysis. For the CD44^+^/CD24^-^ subpopulation analysis, nontreated cells incubated with CD24-PE alone or CD44-FITC alone were used as controls to determine nonspecific signals and gain the gates for CD24^+^ and CD44^+^ subpopulations, respectively. For the ALDH1^+^ subpopulation analysis, diethylaminobenzaldehyde, an ALDH inhibitor, was used as a control for each treatment; for each sample, the cells treated with diethylaminobenzaldehyde were used as a control to gain the gate for ALDH1^+^ subpopulations. To determine the effect of MELK-In-17 on cancer stem-like cell subpopulations, we seeded 3 × 10^5^ cells in 3 mL of complete medium in 60-mm plates and treated the cells the next day with MELK-In-17 at 0, 1, and 2.5 µM. At 48 hours after treatment, cells were harvested and subjected to ALDH1 and CD44^+^/CD24^-^ subpopulation analysis.

**Quantitative reverse transcriptase PCR**

4T1 cells were treated with MELK-In-17 at 0, 0.5, and 2 µM for 24 hours. Following treatment, total RNA was extracted from the cells using the miRNeasy mini kit (Sigma-Aldrich, St. Louis, MO, USA) according to the manufacturer’s instructions. One-step quantitative PCR reactions were performed using the SYBR green quantitative PCR kit (Bio-Rad Laboratories, Hercules, CA, USA) with a pair of murine MELK primers [5’-CTCACCTCGATGAGGATTGCGT-3’ (forward) and 5’-GAAGCAGAAGGTAGGTGGCTGT-3’ (reverse)], murine E-cadherin primers [5’-GGTCATCAGTGTGCTCACCTCT-3’ (forward) and 5’-GCTGTTGTGCTCAAGCCTTCAC-3’ (reverse)], murine N-cadherin primers [5’-CCTCCAGAGTTTACTGCCATGAC-3’ (forward) and 5’-CCACCACTGATTCTGTATGCCG-3’ (reverse)], murine fibronectin primers [5’-CACGGAGGCCACCATTACT-3’ (forward) and 5’-CTTCAGGGCAATGACGTAGAT-3’ (reverse)], murine vimentin primers [5’-CACTAGCCGCAGCCTCTATTC-3’ (forward) and 5’-GTCCACCGAGTCTTGAAGCA-3’ (reverse)], murine β-catenin primers [5’-ACTTGCCACACGTGCAATTC-3’ (forward) and 5’-ATGGTGCGTACAATGGCAGA-3’ (reverse)], murine snail primers [5’-ATTCTCCTGCTCCCACTGC-3’ (forward) and 5’-GACTCTTGGTGCTTGTGGAG-3’ (reverse)], and GAPDH primers [5’- TGTGTCCGTCGTGGATCTGA-3’ (forward) and 5’-TTGCTGTTGAAGTCGCAGGAG-3’ (reverse)]. mRNA of the human housekeeping gene GAPDH was used as a normalization control. mRNA levels of MELK were normalized to the mRNA levels of GAPDH, and the fold induction of MELK mRNA was calculated based on the MELK mRNA level in scramble-treated control cells.

**Differential gene expression profile analysis**

RNA was extracted from parental, Cas9-p15 control, and MELK KO C3 and C28 MDA-MB-231 cells as described above. All experiments were performed in triplicate, generating a total of 24 different gene expression profiles. RNA was hybridized onto Affymetrix Human Transcriptome Arrays (version 2.0). Raw gene expression data were read into R using the BioC-package affy, normalized using the Robust MultiArray (RMA) algorithm, and log_2_-transformed.

Probes with normalized expression values exceeding log_2_(100) in at least 25% of the arrays were filtered for further analysis. In addition, redundancy in probes mapping to the same gene was removed by retaining only the probe with the largest reported variation in gene expression measured by standard deviation. In total, 10,059 unique genes were included for final analysis.

Differences in gene expression were analyzed using generalized linear models (BioC-package limma). The design matrix was set up to only contain the cell type variable (i.e., parental, Cas9-p15, and MEKL KO MDA-MB-231 cells) without intercept term. Genes differentially expressed between all pairs of cell types were identified, and only genes with a False Discovery Rate (FDA) corrected *P* value inferior to 10% were considered significant. Results are represented in volcano plot format. Overlaps between list of differentially expressed genes and between any pair of cell types were quantified using the Jaccard Index (BioC-package GeneOverlap). Vectors of log_2_ fold-changes were subjected to gene set enrichment analysis (GSEA – BioC-package fgsea) for hallmark gene sets (Molecular Signatures Database – Broad Institute). Results were visualized in a modified volcano plot format.

To identify potential regulators of differential gene expression, the BioC-package VIPER was used. The VIPER algorithm virtually infers protein activity levels of both transcription factors and signal transduction proteins based on target gene mRNA expression. It considers the mode of action (i.e., activation or suppression), the regulator-target gene interaction confidence, and the pleiotropic nature of each target gene regulation. The VIPER algorithm was run without a null model and with lists of target genes for 6,053 regulators in a background specific for breast cancer cells (BioC-package aracne.networks). Prior to the VIPER analysis, nominal *P* values resulting from differential gene expression analysis (vide supra) were Z-transformed. Secondary to the core VIPER analysis, a shadow analysis was performed to identify pleiotropic interactions. Regulator proteins with an FDR corrected *P* values inferior to 10% after correction for the shadow effect were subjected to overrepresentation analysis for the WikiPathways and Reactome gene sets using the BioC-package fgsea. Gene sets with an FDR corrected *P* values inferior to 10% were considered significant.

The list of potential regulator proteins was further prioritized using a protein-protein interaction analysis based on the STRING network (https://string-db.org) that was filtered to only contain physical interactions with a minimal confidence score of 0.4 (on a scale of 0 to 1). Hence, the set of potential regulator proteins was limited to only direct MELK interaction partners or proteins that interact with MELK through at most one intermediate protein. The resulting set of prioritized regulator proteins was used to construct a subnetwork that was subjected to louvain clustering (R-package igraph), and resulting clusters were inspected for global differences in protein activation using the VIPER scores.

**References**

1. Strober W. Trypan Blue Exclusion Test of Cell Viability. Curr Protoc Immunol **2015**;111:A3 B 1-A3 B

2. Gloeckner H, Jonuleit T, Lemke HD. Monitoring of cell viability and cell growth in a hollow-fiber bioreactor by use of the dye Alamar Blue. J Immunol Methods **2001**;252:131-8

3. Franken NA, Rodermond HM, Stap J, Haveman J, van Bree C. Clonogenic assay of cells in vitro. Nat Protoc **2006**;1:2315-9

4. Zhang Y, Yu D, Xia W, Hung MC. HER-2/neu-targeting cancer therapy via adenovirus-mediated E1A delivery in an animal model. Oncogene **1995**;10:1947-54
