# Supplementary material for: Maternal Embryonic Leucine Zipper Kinase is Associated with Metastasis in Triple-negative Breast Cancer
Source: Cancer Res Commun. 2023 Jun 20;3(6):1078–92. doi: 10.1158/2767-9764.CRC-22-0330 (PMC10281291; doi:10.1158/2767-9764.CRC-22-0330)
Supplement: Supplementary Figure S1 — Figure S1. Inhibitory effect of treatment with MELK-In-17 on the expression of epithelial-to-mesenchymal transition markers in murine 4T1 triple-negative breast cancer cells. [file crc-22-0330-s02.docx]

**Supplementary Figure S1**
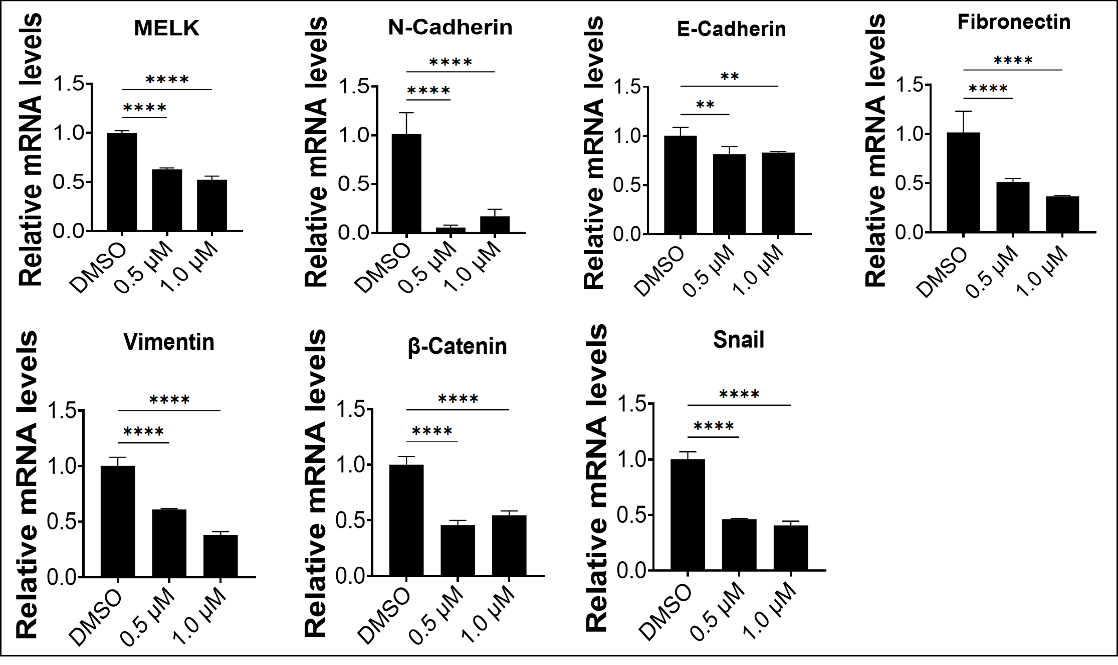


**Figure S1.** **Inhibitory effect of treatment with MELK-In-17 on the expression of epithelial-to-mesenchymal transition markers in murine 4T1 triple-negative breast cancer cells.** 4T1 cells were treated with MELK-In-17 for 24 hours, followed by RNA extraction. mRNA expression levels of epithelial-to-mesenchymal transition markers were determined by quantitative PCR and normalized to β-actin. Data are presented as mean ± standard deviation. ***P* < 0.01; *****P* < 0.0001.
