# Supplementary material for: Maternal Embryonic Leucine Zipper Kinase is Associated with Metastasis in Triple-negative Breast Cancer
Source: Cancer Res Commun. 2023 Jun 20;3(6):1078–92. doi: 10.1158/2767-9764.CRC-22-0330 (PMC10281291; doi:10.1158/2767-9764.CRC-22-0330)
Supplement: Supplementary Figure S2 — Volcano plots show differences in gene expression in parental and Cas9-p15 control or MELK knockout (KO) MDA-MB-231 cells. [file crc-22-0330-s03.docx]

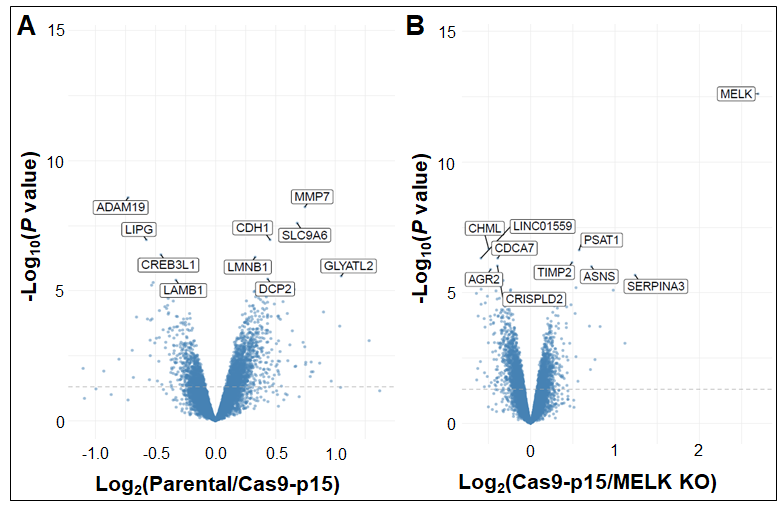
**Supplementary Figure S2**

**Supplementary Figure S2. Volcano plots show differences in gene expression in parental and Cas9-p15 control or MELK knockout (KO) MDA-MB-231 cells.** Differences in gene expression are shown in volcano plot format, where the X-axis denotes the log_2_-transformed fold-change and the Y-axis denotes the –log_10_-transformed *P* value. The log_2_ fold-change is calculated as (**A**) parental/Cas9-p15 control MDA-MB-231 cells and (**B**) parental/MELK KO MDA-MB-231 cells, resulting in positive values for genes overexpressed in the wild-type condition and negative values for genes overexpressed in the KO condition. The nominal *P* value threshold at 5% is indicated by a dashed blue horizontal line. The 10 most differentially expressed genes by *P* value are labelled using their gene symbol.
