# Supplementary material for: Maternal Embryonic Leucine Zipper Kinase is Associated with Metastasis in Triple-negative Breast Cancer
Source: Cancer Res Commun. 2023 Jun 20;3(6):1078–92. doi: 10.1158/2767-9764.CRC-22-0330 (PMC10281291; doi:10.1158/2767-9764.CRC-22-0330)
Supplement: Supplementary Figure S3 — Top 20 master regulators that were differentially expressed in Cas9-p15 control and MELK knockout (KO) MDA-NB-231 cells. [file crc-22-0330-s04.docx]

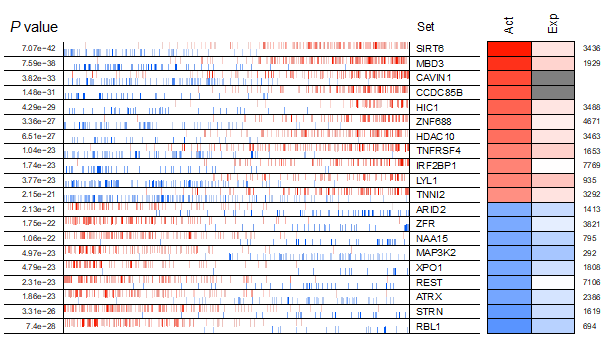
**Supplementary Figure S3**

**Supplementary Figure S3. Top 20 master regulators that were differentially expressed in Cas9-p15 control and MELK knockout (KO) MDA-NB-231 cells.** Based on a breast cancer–specific regulon, differences in gene expression between Cas9-p15 control and MELK KO MDA-MB-231 cells were translated in master regulators of MELK activity in MM231 cells using the VIPER algorithm with correction for pleiotropic interactions. In total, 1,365 master regulators were identified; data for the top 20 master regulators are shown. For each master regulator, the inferred activity score (indicated as Act) and measured expression (indicated as Exp) are shown using a red-blue coloring scheme indicating respectively high and low values in Cas9-p15 control cells. For each master regulator, an enrichment profile is also shown, with the target genes that are positively and negatively regulated by each master regulator indicated by red and blue ticks, respectively, that indicate the position of these target genes in the ordered fold-change Cas9-p15 control, with decreasing expression levels in MELK KO cells relative to Cas9-p15 control from left to right. The *P* value of the enrichment analysis is shown in the first column.
