# Supplementary material for: Maternal Embryonic Leucine Zipper Kinase is Associated with Metastasis in Triple-negative Breast Cancer
Source: Cancer Res Commun. 2023 Jun 20;3(6):1078–92. doi: 10.1158/2767-9764.CRC-22-0330 (PMC10281291; doi:10.1158/2767-9764.CRC-22-0330)
Supplement: Supplementary Figure S4 — Graphical representation of the physical protein-protein interaction network involved in modulating MELK activity in MDA-MB-231 cells. [file crc-22-0330-s05.docx]

**Supplementary Figure S4**
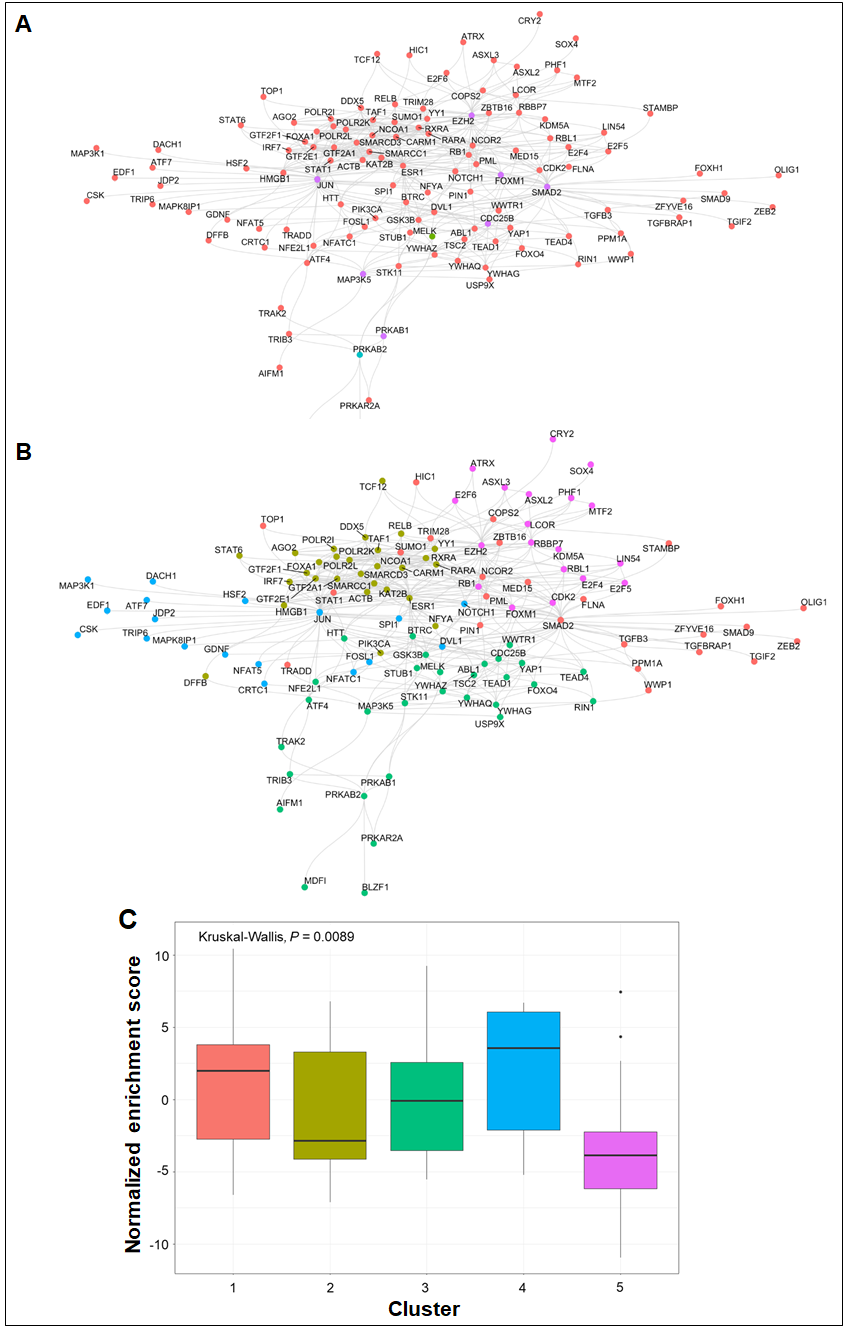


**Supplementary Figure S4. Graphical representation of the physical protein-protein interaction network involved in modulating MELK activity in MDA-MB-231 cells.** **A**, Network showing proteins that have interactions with MELK by at most one intermediate (i.e., 0 or 1). MELK is color-coded in green. PRKAB2, which is color-coded in blue and was identified using the VIPER algorithm, is the only master regulator that has direct interactions with MELK. The proteins color-coded in purple also interact directly with MELK but were not identified using the VIPER algorithm. These are intermediate proteins, identified using the shortest paths algorithm, and these proteins link MELK to all the indirect master regulators. **B**, Network showing proteins, color-coded according to the network module, identified using louvain clustering. **C**, Boxplot depicting the global activity changes for each of the network clusters represented in panel **B**. For each cluster presented along the X-axis, the distribution of the protein activity scores, expressed as a normalized enrichment score of the proteins that belong to that cluster, is shown along the Y-axis. The *P* value of the Kruskal-Wallis test resulting from comparing the distributions between the different clusters is given on the top left. Mean activity scores are indicated by the black horizontal line in each box plot. Scores below zero indicate repression and scores above zero indicate activation upon MELK activation.
