# Supplementary material for: Maternal Embryonic Leucine Zipper Kinase is Associated with Metastasis in Triple-negative Breast Cancer
Source: Cancer Res Commun. 2023 Jun 20;3(6):1078–92. doi: 10.1158/2767-9764.CRC-22-0330 (PMC10281291; doi:10.1158/2767-9764.CRC-22-0330)
Supplement: Supplementary Table S1 — Full list of candidate regulators that were significantly enriched for 60 Reactome and WikiPathways, as identified by Affymetrix Genechip microarray analysis. [file crc-22-0330-s06.docx]

**Supplementary Table S1.** **Full list of candidate regulators that were significantly enriched for 60 Reactome and WikiPathways, as identified by Affymetrix Genechip microarray analysis.**

| **Pathway** | ***P*** | **FDR** | **Overlap** | **No. of genes in pathway** | **Overlapping genes** |
| --- | --- | --- | --- | --- | --- |
| REACTOME_TRANSCRIPTION_OF_THE_HIV_GENOME | 0.000 | 0.000 | 30 | 50 | MNAT1; TAF2; CCNT2; ELOB; ERCC2; POLR2I; ELL; TAF6; GTF2H3; GTF2F1; CCNT1; TAF4; CCNH; GTF2A2; TAF4B; TAF1; POLR2K; TAF5; GTF2E1; GTF2A1; TAF10; POLR2L; TAF7; NELFA; TAF9B; NELFB; SUPT5H; GTF2E2; TAF13; TAF9 |
| REACTOME_HIV_TRANSCRIPTION_INITIATION | 0.000 | 0.000 | 23 | 35 | MNAT1; TAF2; ERCC2; POLR2I; TAF6; GTF2H3; GTF2F1; TAF4; CCNH; GTF2A2; TAF4B; TAF1; POLR2K; TAF5; GTF2E1; GTF2A1; TAF10; POLR2L; TAF7; TAF9B; GTF2E2; TAF13; TAF9 |
| REACTOME_CHROMATIN_MODIFYING_ENZYMES | 0.000 | 0.000 | 74 | 190 | MSL3; ARID4A; HMG20B; ING3; MBD3; KDM5A; EED; ACTB; NFKB2; JADE1; SMARCA2; SMARCD3; REST; NCOA1; ATXN7L3; SETD1A; HDAC10; RBBP7; KAT8; DOT1L; KAT2A; ELP4; SUDS3; BRD8; KAT2B; ATF2; WDR77; ASH1L; DR1; SUPT7L; EPC1; KDM3B; MORF4L2; PRMT1; YEATS4; KDM4B; HAT1; PRMT7; KMT5C; ELP2; CLOCK; KAT7; ACTL6A; SAP130; NCOA2; CARM1; SETDB1; SETD7; OGT; TADA1; SUV39H2; KAT6B; TAF6L; RBBP4; ATXN7; YEATS2; MEAF6; SAP30L; TAF10; CHD3; ATF7IP; SMARCC1; SGF29; DMAP1; SUZ12; EHMT1; SETD2; RUVBL2; H2AX; ARID2; NCOR2; HDAC2; H4C11; TAF9 |
| REACTOME_TRANSCRIPTIONAL_REGULATION_BY_TP53 | 0.000 | 0.000 | 85 | 229 | MNAT1; TAF2; MBD3; TP73; RBL1; CCNT2; BAX; CNOT3; MSH2; RBBP8; RBBP7; TSC2; ELOB; NBN; PPP1R13L; ERCC2; POLR2I; BBC3; ELL; TAF6; RHEB; GTF2H3; CNOT2; RAD50; ATF2; ARID3A; RRAGC; STK11; CDK2; AGO2; CNOT1; GTF2F1; PRMT1; PIN1; E2F8; CCNT1; TAF4; PRKAB2; PRKAA1; YWHAQ; CCNH; MDM2; LRPPRC; BARD1; PML; TAF4B; CARM1; CNOT9; CCNA2; TAF1; POLR2K; TAF5; RAD17; CNOT8; ZNF385A; RBBP4; MEAF6; RFC4; YWHAZ; TSC1; E2F7; TAF10; MLST8; CHD3; YWHAG; TRIAP1; CYCS; BANP; TNFRSF10C; ATR; PIDD1; POLR2L; TAF7; EHMT1; NPM1; NELFA; TAF9B; NOC2L; NELFB; SUPT5H; HDAC2; TAF13; MDM4; E2F4; TAF9 |
| REACTOME_HIV_LIFE_CYCLE | 0.000 | 0.000 | 36 | 73 | MNAT1; TAF2; CCNT2; XPO1; RANGAP1; ELOB; ERCC2; POLR2I; ELL; TAF6; GTF2H3; GTF2F1; CCNT1; TAF4; CCNH; GTF2A2; TAF4B; TAF1; POLR2K; TAF5; RANBP2; GTF2E1; PSIP1; GTF2A1; TAF10; FEN1; POLR2L; TAF7; NELFA; TAF9B; NELFB; SUPT5H; XRCC6; GTF2E2; TAF13; TAF9 |
| REACTOME_HIV_INFECTION | 0.000 | 0.000 | 40 | 88 | MNAT1; TAF2; CCNT2; XPO1; RANGAP1; ELOB; ERCC2; POLR2I; ELL; TAF6; GTF2H3; GTF2F1; CCNT1; TAF4; CCNH; GTF2A2; TAF4B; TAF1; POLR2K; TAF5; RANBP2; GTF2E1; PSIP1; GTF2A1; PSMC3; BTRC; TAF10; B2M; FEN1; POLR2L; TAF7; NPM1; NELFA; TAF9B; NELFB; SUPT5H; XRCC6; GTF2E2; TAF13; TAF9 |
| REACTOME_HATS_ACETYLATE_HISTONES | 0.000 | 0.002 | 38 | 87 | MSL3; ING3; ACTB; JADE1; NCOA1; ATXN7L3; RBBP7; KAT8; KAT2A; ELP4; BRD8; KAT2B; ATF2; DR1; SUPT7L; EPC1; MORF4L2; YEATS4; HAT1; ELP2; CLOCK; KAT7; ACTL6A; SAP130; NCOA2; OGT; TADA1; KAT6B; TAF6L; ATXN7; YEATS2; MEAF6; TAF10; SGF29; DMAP1; RUVBL2; H4C11; TAF9 |
| REACTOME_RNA_POLYMERASE_II_TRANSCRIBES_SNRNA_GENES | 0.000 | 0.003 | 20 | 36 | CCNT2; SNAPC2; POLR2I; ELL; TAF6; ELL2; GTF2F1; CCNT1; GTF2A2; ZC3H8; POLR2K; TAF5; GTF2E1; GTF2A1; SNAPC4; POLR2L; SUPT5H; GTF2E2; TAF13; TAF9 |
| WP_EUKARYOTIC_TRANSCRIPTION_INITIATION | 0.000 | 0.004 | 15 | 24 | POLR2K; MNAT1; GTF2E2; GTF2E1; ERCC2; POLR2I; GTF2H3; TAF9; GTF2H2C; TAF13; TAF6; TAF5; CCNH; GTF2A2; TAF7 |
| REACTOME_TRANSCRIPTIONAL_REGULATION_BY_E2F6 | 0.000 | 0.010 | 16 | 28 | YAF2; EED; RBBP8; RBBP7; EPC1; RNF2; CBX3; PCGF6; RBBP4; RYBP; BMI1; E2F6; MGA; SUZ12; EHMT1; RING1 |
| REACTOME_SIGNALING_BY_RHO_GTPASES_MIRO_GTPASES_AND_RHOBTB3 | 0.000 | 0.028 | 113 | 374 | RHOBTB2; NISCH; FAM13B; ARHGAP31; CUL3; PPP1R12A; MYO9A; ROCK1; ACTB; ARHGEF1; SENP1; NDC80; XPO1; ABL1; MYO9B; SH3BP1; RANGAP1; FGD1; CENPI; MAPK3; CSK; RHOV; CDC37; WASL; LIMK1; DVL1; DNMBP; ARHGAP21; PFN1; ARHGEF17; SRF; ERBIN; ECT2; PLXNA1; RTKN; SOS1; TRAK2; DOCK7; CENPF; TMPO; PIK3CA; CENPK; USP9X; TRIP10; PIN1; CDC42EP1; BAIAP2L2; ARHGAP22; PAK4; DLG4; VAV3; YWHAQ; KIDINS220; ROCK2; ARHGEF4; TEX2; FRS3; NCOA2; RHOT2; EPHA2; ITGB3BP; TPM3; RHOB; PIK3R1; IQGAP2; ARHGAP39; RANBP2; MSI2; OBSCN; SH3RF1; RHOC; RNF20; BUB1B; NCK1; RHPN1; VAV2; TAOK1; PMF1; RACGAP1; YWHAZ; VCP; ARHGAP12; ARHGAP42; COPS2; FRS2; CDC42EP5; AR; GPS1; YWHAG; RND1; CFL1; MAP3K11; SCAI; JUP; RALGAPA1; BAIAP2; RHOG; CAVIN1; ARHGAP45; SCRIB; NF2; ARAP1; H2AX; EVL; MRTFA; ZNF512B; FLNA; SRGAP1; H4C11; PPP1CB; TAX1BP3; ARHGEF25; ARHGDIG |
| REACTOME_FORMATION_OF_RNA_POL_II_ELONGATION_COMPLEX | 0.000 | 0.029 | 19 | 39 | MNAT1; AFF4; CCNT2; ELOB; ERCC2; POLR2I; ELL; GTF2H3; GTF2F1; CCNT1; MLLT1; CCNH; EAF1; POLR2K; MLLT3; POLR2L; NELFA; NELFB; SUPT5H |
| REACTOME_CELL_CYCLE | 0.000 | 0.029 | 77 | 239 | E2F2; CDK11A; MNAT1; RFC1; PPP1R12A; POLD1; MCM6; RBL1; NDC80; GSK3B; XPO1; ATRX; ABL1; MCM5; RANGAP1; RBBP8; STAG2; RBBP7; CENPI; MAPK3; NBN; POLR2I; E2F3; RAD50; ORC2; SUMO1; BLZF1; CENPF; TMPO; CENPK; CDK2; MCM8; AKAP9; TOP2A; E2F5; YWHAQ; CCNH; MDM2; RAB1A; BARD1; RB1; ITGB3BP; EML4; CCNA2; TERF1; POLR2K; RAD17; SMARCA5; RANBP2; BUB1B; TAOK1; PMF1; ZNF385A; RBBP4; RFC4; RAD21; YWHAZ; PSMC3; BTRC; FEN1; E2F6; YWHAG; RAB1B; ATR; POLR2L; UBE2N; NPM1; RUVBL2; BRCC3; H2AX; LIN54; H4C11; MDM4; CEP290; E2F4; PPP1CB; CDK11B |
| REACTOME_FORMATION_OF_THE_EARLY_ELONGATION_COMPLEX | 0.001 | 0.042 | 11 | 19 | MNAT1; ERCC2; POLR2I; GTF2H3; GTF2F1; CCNH; POLR2K; POLR2L; NELFA; NELFB; SUPT5H |
| REACTOME_NOTCH_HLH_TRANSCRIPTION_PATHWAY | 0.000 | 0.029 | 15 | 28 | HDAC9; HDAC7; NOTCH3; HDAC6; HDAC10; KAT2A; HDAC5; KAT2B; NOTCH2; NOTCH1; HDAC11; MAML2; NCOR2; HDAC2; NOTCH4 |
| REACTOME_DNA_REPAIR | 0.000 | 0.029 | 52 | 149 | PPP5C; MNAT1; RFC1; POLD1; ACTB; SIRT6; PPIE; MSH2; ABL1; YY1; RBBP8; NBN; ERCC2; POLR2I; TFPT; ELL; GTF2H3; TIMELESS; ASCC3; MSH3; RAD50; FANCL; SUMO1; MSH6; UBE2B; CDK2; KDM4B; MUTYH; CCNH; ACTL6A; BARD1; TDG; CCNA2; TERF1; POLR2K; PPP4C; RAD17; SMARCA5; RFC4; VCP; COPS2; APBB1; FEN1; GPS1; ATR; POLR2L; UBE2N; BRCC3; H2AX; XRCC6; H4C11; PRKDC |
| REACTOME_RNA_POLYMERASE_I_TRANSCRIPTION_INITIATION | 0.000 | 0.029 | 16 | 31 | MNAT1; MBD3; RRN3; RBBP7; ERCC2; KAT2A; GTF2H3; KAT2B; TTF1; CCNH; POLR2K; RBBP4; TAF1D; CHD3; POLR2L; HDAC2 |
| REACTOME_METABOLISM_OF_RNA | 0.000 | 0.029 | 43 | 118 | MNAT1; CCAR1; YBX1; THOC1; DDX1; XPO1; PPIE; CNOT3; CDC5L; PRPF6; PQBP1; ERCC2; POLR2I; HSPB1; DDX5; GTF2H3; CNOT2; SRSF3; SF3B1; WDR77; CNOT1; GTF2F1; CCNH; TGS1; WDR12; HNRNPD; ZCRB1; CNOT9; POLR2K; RANBP2; CNOT8; TNFSF13; RPS14; YWHAZ; PSMC3; PLRG1; POLR2L; UTP11; TBL3; ZFP36L1; PNRC2; SUPT5H; C1D |
| REACTOME_POST_TRANSLATIONAL_PROTEIN_MODIFICATION | 0.000 | 0.029 | 132 | 453 | NR1H3; CUL3; RAB27B; HDAC7; ASB1; SP100; FSTL3; ACTB; NFKB2; SENP1; KEAP1; RAB21; NCOA1; RAB10; MUL1; ESR1; LGALS1; RANGAP1; YY1; ARFGAP1; STAG2; RBBP7; AXIN1; ELOB; RIPK2; TFPT; FKBP8; FBXW4; DDX5; KAT2A; RAB5C; WSB1; RAB34; WFS1; ZBTB16; CAND1; RAB5B; SPSB2; SUDS3; KAT2B; ASB3; SUMO1; PARK7; FBXO6; UBE2B; IDE; RNF2; NEURL2; STAMBP; USP9X; RAB17; THRA; TRAF2; RAB2B; APOE; TRIM28; NR1H2; TOP2A; RARA; APC; MDM2; ACTL6A; USP20; RAB30; RAB1A; PREB; BARD1; TDG; NCOA2; PML; RAB40B; CBX8; CBX4; PEX14; WDTC1; DCAF6; ASXL2; CCNA2; RAB19; OGT; RANBP2; THY1; RNF20; USP16; ASB16; SPSB3; MYSM1; TGFBR2; ATXN7; SHISA5; RAD21; VCP; PSMC3; RAB8B; BTRC; COPS2; TAF10; RAB4B; RAB26; BMI1; TNIP2; AR; NSMCE1; GPS1; CHD3; SOCS5; RAB43; RAB1B; WSB2; HIC1; UBE2N; SUZ12; NPM1; FOXO4; NSMCE3; RAB11B; BRCC3; RXRA; PPARA; TAF9B; NCOR2; MRTFA; HDAC2; H4C11; RAB40C; MDM4; TOP1; RING1; ZBED1; ASB14; PRKDC; IKBKG |
| REACTOME_MRNA_SPLICING_MINOR_PATHWAY | 0.001 | 0.035 | 8 | 11 | YBX1; PRPF6; POLR2I; SF3B1; GTF2F1; ZCRB1; POLR2K; POLR2L |
| REACTOME_REGULATION_OF_PTEN_GENE_TRANSCRIPTION | 0.001 | 0.035 | 22 | 50 | HDAC7; MBD3; EED; REST; RBBP7; MAPK3; RHEB; HDAC5; ATN1; ATF2; RRAGC; RNF2; CBX8; CBX4; RBBP4; MLST8; BMI1; CHD3; SUZ12; MAF1; HDAC2; RING1 |
| REACTOME_CELLULAR_RESPONSES_TO_STIMULI | 0.001 | 0.035 | 88 | 286 | NFYA; E2F2; CUL3; EED; KEAP1; SMARCD3; GSK3B; XPO1; NCOA1; PPP1R15A; HDAC6; CABIN1; XBP1; ARFGAP1; TRIB3; RBBP7; MAPK3; ELOB; NBN; CRTC1; ERF; HSPB1; RHEB; WFS1; CRYAB; PTGES3; ASF1A; E2F3; RAD50; ATF2; MEF2D; ZBTB17; RRAGC; RPS6KA1; CREB1; NFYB; RNF2; CDK2; SIN3B; ATF4; CLOCK; ETS1; MDM2; EGLN1; TGS1; PREB; LRPPRC; RB1; NCOA2; CRTC3; MINK1; CBX8; CBX4; SOD1; CARM1; CCNA2; TERF1; VENTX; RANBP2; TNIK; FLCN; CXXC1; BACH1; RBBP4; CREBRF; RPS14; VCP; PRDX3; PSMC3; MLST8; BMI1; AR; EIF2AK3; CYCS; ATR; SUZ12; EHMT1; HSF1; RXRA; PPARA; H2AX; NCOR2; H4C11; ERO1A; MAFK; MDM4; RING1; GPX1 |
| REACTOME_TP53_REGULATES_TRANSCRIPTION_OF_CELL_CYCLE_GENES | 0.001 | 0.035 | 17 | 35 | RBL1; BAX; CNOT3; CNOT2; ARID3A; CDK2; CNOT1; PRMT1; E2F8; CARM1; CNOT9; CCNA2; CNOT8; ZNF385A; E2F7; NPM1; E2F4 |
| REACTOME_RHO_GTPASES_ACTIVATE_FORMINS | 0.001 | 0.035 | 20 | 44 | ACTB; NDC80; XPO1; RANGAP1; CENPI; DVL1; PFN1; SRF; CENPF; CENPK; ITGB3BP; RHOB; RANBP2; RHOC; BUB1B; TAOK1; PMF1; SCAI; EVL; MRTFA |
| REACTOME_RHO_GTPASE_EFFECTORS | 0.001 | 0.036 | 47 | 135 | PPP1R12A; ROCK1; ACTB; NDC80; XPO1; ABL1; RANGAP1; CENPI; MAPK3; WASL; LIMK1; DVL1; PFN1; SRF; RTKN; CENPF; CENPK; PIN1; DLG4; YWHAQ; ROCK2; NCOA2; ITGB3BP; RHOB; IQGAP2; RANBP2; RHOC; BUB1B; NCK1; RHPN1; TAOK1; PMF1; YWHAZ; AR; YWHAG; CFL1; SCAI; BAIAP2; RHOG; NF2; H2AX; EVL; MRTFA; FLNA; H4C11; PPP1CB; TAX1BP3 |
| REACTOME_RNA_POLYMERASE_II_TRANSCRIPTION | 0.001 | 0.036 | 254 | 954 | NFYA; ZNF263; TEAD3; YAF2; WWTR1; MNAT1; NR1H3; HDAC9; HDAC7; TAF2; SPI1; CBFB; TCF3; MBD3; AFF4; NOTCH3; TEAD2; EED; TP73; THOC1; SMARCA2; RBL1; SMARCD3; CCNT2; GSK3B; ZNF446; ZNF213; BAX; CNOT3; ESR1; HDAC6; MSH2; ABL1; SETD1A; MED15; HDAC10; PPM1A; YY1; RBBP8; RBBP7; MAPK3; AXIN1; TSC2; ZNF500; STUB1; ELOB; NBN; PPP1R13L; ERCC2; MED25; SNAPC2; MED26; POLR2I; BBC3; ELL; TAF6; RHEB; MED13; KAT2A; COL1A1; HDAC5; GTF2H3; CNOT2; SRSF3; CCNC; RAD50; KAT2B; STAT1; ATF2; ARID3A; SUMO1; TFAP2E; RRAGC; STK11; CREB1; TGIF2; ELL2; EPC1; ELF1; NFYB; ZNF706; RNF2; ZNF205; CBX3; WWP1; CDK2; NFE2; AGO2; USP9X; MED20; DEK; CNOT1; GTF2F1; ZNF133; CITED1; THRA; PRMT1; ZNF484; YEATS4; PIN1; SIN3B; E2F8; CCNT1; APOE; MLLT1; TAF4; TRIM28; NR1H2; RARA; PRKAB2; PRKAA1; CDK8; E2F5; NOTCH2; YWHAQ; CCNH; MDM2; ACTL6A; YAP1; LRPPRC; BARD1; RB1; TCF12; GTF2A2; PML; TAF4B; CBX8; CBX4; ERBB2; ZFP14; SIRT3; CARM1; ZC3H8; HSPD1; CNOT9; EAF1; CCNA2; TAF1; POLR2K; NOTCH1; TAF5; FOXO1; VENTX; TCF7L1; RAD17; GTF2E1; CNOT8; PCGF6; ATAD2; PINK1; ZNF714; MED27; ZNF385A; RBBP4; HDAC11; RYBP; MEAF6; RFC4; YWHAZ; GTF2A1; SNAPC4; TSC1; E2F7; PSMC3; TAF10; ZNF226; ZNF668; MLST8; BMI1; E2F6; AR; ZNF354A; ZNF747; CHD3; YWHAG; TRIAP1; ZNF692; ZNF562; MLLT3; CYCS; BANP; ESRRA; SMARCC1; TNFRSF10C; ZNF791; MGA; NR1D2; ATR; ZNF519; NR2C2; PIDD1; POLR2L; ZNF619; ZNF354B; SUZ12; TAF7; ZNF664; PTPN11; CITED4; MED14; PCGF5; EHMT1; ZNF707; NPM1; ZNF703; IRAK1; MAML2; FOXO4; MED12; NELFA; ZNF267; RXRA; PPARA; TEAD1; ZNF546; TAF9B; ZFP69B; H2AX; NOC2L; NELFB; ZNF567; ARID2; ZNF699; SUPT5H; PTPN1; ZNF777; ZNF775; ZNF605; NCOR2; HDAC2; ZNF785; H4C11; GTF2E2; ZNF720; ZNF517; ZNF860; HTT; TAF13; TEAD4; ZNF71; ZNF770; ZNF485; ZKSCAN8; ZNF26; ZNF480; ZNF28; MDM4; RING1; RXRB; NOTCH4; E2F4; ZNF492; ZNF688; TAF9 |
| REACTOME_HIV_TRANSCRIPTION_ELONGATION | 0.001 | 0.040 | 14 | 27 | MNAT1; ELOB; ERCC2; POLR2I; ELL; GTF2H3; GTF2F1; CCNT1; CCNH; POLR2K; POLR2L; NELFA; NELFB; SUPT5H |
| REACTOME_MRNA_SPLICING | 0.001 | 0.042 | 16 | 33 | CCAR1; YBX1; PPIE; CDC5L; PRPF6; PQBP1; POLR2I; DDX5; SRSF3; SF3B1; GTF2F1; HNRNPD; ZCRB1; POLR2K; PLRG1; POLR2L |
| WP_CELL_CYCLE | 0.001 | 0.046 | 27 | 68 | TGFB3; MCM5; MCM6; RBL1; ABL1; CCNA2; MDM2; E2F5; GADD45G; E2F4; E2F3; E2F2; ATR; ORC2; CDK2; STAG2; YWHAQ; PRKDC; YWHAG; YWHAZ; HDAC2; TTK; ZBTB17; GSK3B; RAD21; CCNH; RB1 |
| REACTOME_EPIGENETIC_REGULATION_OF_GENE_EXPRESSION | 0.001 | 0.052 | 33 | 89 | MNAT1; MBD3; EED; ACTB; GSK3B; RBBP7; ERCC2; KAT2A; GTF2H3; SUDS3; PHF1; KAT2B; SF3B1; CBX3; DEK; TTF1; SIN3B; CCNH; SAP130; TET1; TDG; MTF2; POLR2K; SMARCA5; RBBP4; SAP30L; TAF1D; CHD3; POLR2L; SUZ12; H2AX; HDAC2; H4C11 |
| REACTOME_TRANSCRIPTIONAL_ACTIVATION_OF_MITOCHONDRIAL_BIOGENESIS | 0.001 | 0.052 | 19 | 43 | SMARCD3; NCOA1; GABPB1; CRTC1; TFAM; ATF2; MEF2D; CREB1; TGS1; NCOA2; CRTC3; SIRT3; CARM1; TFB2M; CYCS; ESRRA; IDH2; RXRA; PPARA |
| WP_MRNA_PROCESSING | 0.001 | 0.052 | 14 | 28 | RBM39; NONO; PRPF6; SF3B1; YBX1; SFPQ; PRMT1; PRMT2; SFSWAP; SUPT5H; DDX1; SRSF3; HNRNPD; SRPK1 |
| WP_ETHANOL_EFFECTS_ON_HISTONE_MODIFICATIONS | 0.001 | 0.052 | 10 | 17 | KAT2B; HDAC2; HDAC10; HDAC7; HDAC6; HDAC5; HAT1; HDAC9; ADH1A; ATF2 |
| WP_VEGFAVEGFR2_SIGNALING_PATHWAY | 0.001 | 0.056 | 78 | 255 | FOXO1; LDB2; FLII; NOTCH4; GIPC1; IDH2; MAP2K2; NFATC1; CREB1; HSPB1; RND1; ASCC3; ITGB5; ETS1; ATF2; ITGAV; PBXIP1; ELK1; SRF; FOXO4; PTPN1; CBL; SRPK1; NCK1; PTPN11; PTPN14; MMP14; HDAC5; SHC2; INPP4B; CFL1; CLIC1; ZC3H15; PRKAA1; STAT1; HRAS; HLX; ICAM1; MLST8; HMGB1; STAT6; TNFRSF25; PRKCI; PLCG1; PFN1; MAPK3; VAV2; BSG; CYCS; HDAC9; ATF4; SMARCA2; TNFRSF10C; ABL1; MDM2; PIK3R1; PIK3CA; RHOC; TEAD4; HDAC7; BCAR1; QKI; TRIP4; TFAM; EIF2AK3; GSK3B; EPHA2; TAOK2; RBM39; ROCK2; FRS2; TPM3; ROCK1; GPX1; RAP1B; DOK1; CSK; LIMK1 |
| REACTOME_HIV_ELONGATION_ARREST_AND_RECOVERY | 0.002 | 0.057 | 11 | 20 | CCNT2; ELOB; POLR2I; ELL; GTF2F1; CCNT1; POLR2K; POLR2L; NELFA; NELFB; SUPT5H |
| REACTOME_CHROMOSOME_MAINTENANCE | 0.002 | 0.057 | 21 | 50 | RFC1; POLD1; ATRX; RBBP7; CENPI; POLR2I; CENPK; CDK2; ITGB3BP; CCNA2; TERF1; POLR2K; SMARCA5; RBBP4; RFC4; FEN1; POLR2L; NPM1; RUVBL2; H2AX; H4C11 |
| REACTOME_REGULATION_OF_TP53_ACTIVITY_THROUGH_PHOSPHORYLATION | 0.002 | 0.061 | 27 | 70 | TAF2; RBBP8; NBN; TAF6; RAD50; STK11; CDK2; PIN1; TAF4; PRKAB2; PRKAA1; MDM2; BARD1; TAF4B; CCNA2; TAF1; TAF5; RAD17; RFC4; TAF10; ATR; TAF7; TAF9B; NOC2L; TAF13; MDM4; TAF9 |
| REACTOME_ABORTIVE_ELONGATION_OF_HIV_1_TRANSCRIPT_IN_THE_ABSENCE_OF_TAT | 0.002 | 0.062 | 7 | 10 | POLR2I; GTF2F1; POLR2K; POLR2L; NELFA; NELFB; SUPT5H |
| REACTOME_PROCESSING_OF_CAPPED_INTRON_CONTAINING_PRE_MRNA | 0.002 | 0.062 | 18 | 41 | CCAR1; YBX1; THOC1; PPIE; CDC5L; PRPF6; PQBP1; POLR2I; DDX5; SRSF3; SF3B1; GTF2F1; HNRNPD; ZCRB1; POLR2K; RANBP2; PLRG1; POLR2L |
| REACTOME_MRNA_CAPPING | 0.002 | 0.062 | 9 | 15 | MNAT1; ERCC2; POLR2I; GTF2H3; GTF2F1; CCNH; POLR2K; POLR2L; SUPT5H |
| WP_DNA_REPAIR_PATHWAYS_FULL_NETWORK | 0.002 | 0.063 | 22 | 54 | MNAT1; XRCC6; POLD1; TDG; MSH6; MUTYH; H2AX; RAD50; GTF2H3; ATR; HMGB1; RFC1; FANCL; ERCC2; PRKDC; GTF2H2C; FEN1; RFC4; NBN; MSH2; CCNH; MSH3 |
| REACTOME_NUCLEOTIDE_EXCISION_REPAIR | 0.002 | 0.071 | 20 | 48 | MNAT1; RFC1; POLD1; ACTB; PPIE; YY1; ERCC2; POLR2I; TFPT; ELL; GTF2H3; SUMO1; CCNH; ACTL6A; POLR2K; RFC4; COPS2; GPS1; POLR2L; UBE2N |
| REACTOME_ENERGY_DEPENDENT_REGULATION_OF_MTOR_BY_LKB1_AMPK | 0.002 | 0.071 | 10 | 18 | STRADB; PPM1A; TSC2; RHEB; RRAGC; STK11; PRKAB2; PRKAA1; TSC1; MLST8 |
| REACTOME_SIGNALING_BY_ALK_IN_CANCER | 0.003 | 0.080 | 17 | 39 | MAPK3; STRN; BCL11A; PIK3CA; PLCG1; RRBP1; FRS3; TPM3; EML4; PIK3R1; MSN; RANBP2; SQSTM1; FRS2; EIF2AK3; RNF213; NPM1 |
| REACTOME_RHO_GTPASES_ACTIVATE_ROCKS | 0.003 | 0.080 | 8 | 13 | PPP1R12A; ROCK1; LIMK1; ROCK2; RHOB; RHOC; CFL1; PPP1CB |
| WP_ATM_SIGNALING_IN_DEVELOPMENT_AND_DISEASE | 0.003 | 0.082 | 15 | 33 | PRKAA1; STK11; HSPB1; RNF20; H2AX; TRIM28; PPP5C; RAD50; RBBP8; PRKDC; NBN; ATR; ATF2; IKBKG; TSC2 |
| WP_LEPTIN_SIGNALING_PATHWAY | 0.003 | 0.084 | 26 | 69 | PTPN11; CREB1; RPS6KA1; KHDRBS1; PLCG1; NCOA1; FOXO1; ROCK2; MAPK3; PRKAA1; STAT1; SOS1; ESR1; HRAS; ROCK1; BAD; ELK1; PIK3R1; PTPN1; ERBB2; BAX; GSK3B; SH2B1; IKBKG; REL; MAP2K2 |
| REACTOME_REGULATION_OF_TP53_ACTIVITY | 0.003 | 0.091 | 40 | 119 | TAF2; MBD3; TP73; RBBP8; RBBP7; NBN; PPP1R13L; TAF6; RAD50; STK11; CDK2; PIN1; TAF4; PRKAB2; PRKAA1; MDM2; BARD1; PML; TAF4B; CCNA2; TAF1; TAF5; RAD17; ZNF385A; RBBP4; MEAF6; RFC4; TAF10; MLST8; CHD3; BANP; ATR; TAF7; EHMT1; TAF9B; NOC2L; HDAC2; TAF13; MDM4; TAF9 |
| REACTOME_RNA_POLYMERASE_I_TRANSCRIPTION_TERMINATION | 0.004 | 0.091 | 9 | 16 | MNAT1; ERCC2; GTF2H3; TTF1; CCNH; POLR2K; TAF1D; CAVIN1; POLR2L |
| REACTOME_SUMOYLATION | 0.004 | 0.091 | 44 | 134 | NR1H3; HDAC7; SP100; NFKB2; SENP1; NCOA1; ESR1; RANGAP1; STAG2; DDX5; SUMO1; PARK7; RNF2; THRA; TRIM28; NR1H2; TOP2A; RARA; MDM2; TDG; NCOA2; PML; CBX8; CBX4; RANBP2; RAD21; BMI1; AR; NSMCE1; CHD3; HIC1; SUZ12; NPM1; NSMCE3; RXRA; PPARA; NCOR2; MRTFA; HDAC2; H4C11; TOP1; RING1; ZBED1; IKBKG |
| REACTOME_S_PHASE | 0.004 | 0.091 | 21 | 53 | MNAT1; RFC1; POLD1; MCM6; GSK3B; MCM5; STAG2; ORC2; CDK2; MCM8; E2F5; CCNH; RB1; CCNA2; RBBP4; RFC4; RAD21; PSMC3; FEN1; LIN54; E2F4 |
| REACTOME_CYTOSOLIC_SENSORS_OF_PATHOGEN_ASSOCIATED_DNA | 0.004 | 0.091 | 17 | 40 | NFKB2; NFKBIB; POLR3G; LRRFIP1; IRF3; POLR3F; POLR2K; IFI16; TLR3; STAT6; POLR2L; TBK1; IRF7; XRCC6; NKIRAS1; PRKDC; IKBKG |
| WP_ENERGY_METABOLISM | 0.004 | 0.091 | 17 | 40 | RXRA; PRKAA1; PPARA; PRKAB2; CREB1; TFB2M; MEF2B; ESRRA; TFAM; NCOA1; MEF2A; FOXO1; MEF2D; PRMT1; SIRT3; GSK3B; ATF2 |
| REACTOME_PTEN_REGULATION | 0.004 | 0.091 | 26 | 70 | HDAC7; MBD3; EED; REST; RBBP7; MAPK3; STUB1; RHEB; HDAC5; ATN1; ATF2; RRAGC; RNF2; AGO2; PML; CBX8; CBX4; RBBP4; PSMC3; MLST8; BMI1; CHD3; SUZ12; MAF1; HDAC2; RING1 |
| REACTOME_ESTROGEN_DEPENDENT_GENE_EXPRESSION | 0.004 | 0.091 | 30 | 84 | CBFB; NCOA1; ESR1; YY1; STAG2; AXIN1; POLR2I; DDX5; PTGES3; KAT2B; ATF2; AGO2; GTF2F1; CITED1; PRMT1; KDM4B; CCNT1; FOXA1; GTF2A2; TLE3; NCOA2; CARM1; POLR2K; CHD1; RAD21; GTF2A1; CXXC5; POLR2L; H2AX; H4C11 |
| REACTOME_SIGNALING_BY_FGFR4_IN_DISEASE | 0.004 | 0.091 | 7 | 11 | SOS1; PIK3CA; PLCG1; PIK3R1; FGFR4; FRS2; HRAS |
| WP_MIR5093P_ALTERATION_OF_YAP1ECM_AXIS | 0.004 | 0.091 | 7 | 11 | BCAR1; TEAD2; TEAD4; TEAD1; TEAD3; COL1A1; YAP1 |
| REACTOME_ESR_MEDIATED_SIGNALING | 0.004 | 0.093 | 47 | 146 | PPP5C; CBFB; XPO1; NCOA1; ESR1; YY1; STAG2; MAPK3; AXIN1; POLR2I; HSPB1; DDX5; PTGES3; SRF; KAT2B; GNAI2; STRN; ATF2; CREB1; PIK3CA; AGO2; GTF2F1; CITED1; PRMT1; ELK1; GNG13; KDM4B; CCNT1; FOXA1; GTF2A2; TLE3; NCOA2; IGF1R; CARM1; PIK3R1; POLR2K; CHD1; RAD21; GTF2A1; CXXC5; GNB2; GNG12; HRAS; SPHK1; POLR2L; H2AX; H4C11 |
| REACTOME_REGULATION_OF_LIPID_METABOLISM_BY_PPARALPHA | 0.004 | 0.093 | 33 | 95 | NFYA; NR1H3; SMARCD3; NCOA1; MED15; TRIB3; MED25; MED26; AHR; MED13; CCNC; NFYB; MED13L; MED20; SIN3B; NR1H2; CDK8; GRHL1; CLOCK; TGS1; NCOA2; CARM1; MED22; MED21; MED27; MED11; ESRRA; MED14; MED12; RXRA; PPARA; NCOR2; RXRB |
| REACTOME_RHOV_GTPASE_CYCLE | 0.004 | 0.094 | 13 | 28 | MYO9A; RHOV; WASL; USP9X; PAK4; EPHA2; TPM3; PIK3R1; SH3RF1; NCK1; ARHGAP12; MAP3K11; ZNF512B |

Abbreviations: FDR, false discovery rate.
